# NusG prevents transcriptional invasion of H-NS-silenced genes

**DOI:** 10.1371/journal.pgen.1008425

**Published:** 2019-10-07

**Authors:** Lionello Bossi, Mathilde Ratel, Camille Laurent, Patricia Kerboriou, Andrew Camilli, Eric Eveno, Marc Boudvillain, Nara Figueroa-Bossi

**Affiliations:** 1 Institute for Integrative Biology of the Cell (I2BC), CEA, CNRS, Université Paris-Sud, Université Paris-Saclay, France; 2 Department of Molecular Biology and Microbiology, Tufts University, Boston, MA, United States of America; 3 Centre de Biophysique Moléculaire, CNRS UPR4301, rue Charles Sadron, France; University of Wisconsin-Madison, UNITED STATES

## Abstract

Evolutionarily conserved NusG protein enhances bacterial RNA polymerase processivity but can also promote transcription termination by binding to, and stimulating the activity of, Rho factor. Rho terminates transcription upon anchoring to cytidine-rich motifs, the so-called Rho utilization sites (Rut) in nascent RNA. Both NusG and Rho have been implicated in the silencing of horizontally-acquired A/T-rich DNA by nucleoid structuring protein H-NS. However, the relative roles of the two proteins in H-NS-mediated gene silencing remain incompletely defined. In the present study, a *Salmonella* strain carrying the *nusG* gene under the control of an arabinose-inducible repressor was used to assess the genome-wide response to NusG depletion. Results from two complementary approaches, *i*) screening *lacZ* protein fusions generated by random transposition and *ii*) transcriptomic analysis, converged to show that loss of NusG causes massive upregulation of *Salmonella* pathogenicity islands (SPIs) and other H-NS-silenced loci. A similar, although not identical, SPI-upregulated profile was observed in a strain with a mutation in the *rho* gene, Rho K130Q. Surprisingly, Rho mutation Y80C, which affects Rho’s primary RNA binding domain, had either no effect or made H-NS-mediated silencing of SPIs even tighter. Thus, while corroborating the notion that bound H-NS can trigger Rho-dependent transcription termination in vivo, these data suggest that H-NS-elicited termination occurs entirely through a NusG-dependent pathway and is less dependent on Rut site binding by Rho. We provide evidence that through Rho recruitment, and possibly through other still unidentified mechanisms, NusG prevents pervasive transcripts from elongating into H-NS-silenced regions. Failure to perform this function causes the feedforward activation of the entire *Salmonella* virulence program. These findings provide further insight into NusG/Rho contribution in H-NS-mediated gene silencing and underscore the importance of this contribution for the proper functioning of a global regulatory response in growing bacteria. The complete set of transcriptomic data is freely available for viewing through a user-friendly genome browser interface.

## Author summary

Most virulence genes of pathogenic Enterobacteria lie within genomic islands acquired through horizontal transfer in the course of evolution. While essential during infection, expression of many of these genes is detrimental for growth outside the host. Hence, mechanisms have evolved that prevent transcription of this foreign DNA, except when needed. A major mechanism uses Histone-like protein H-NS which binds to AT-rich nucleation sites, a hallmark of horizontally acquired DNA, forming oligomeric structures that silence transcription over extended regions. This silencing activity is all the more important as A/T-rich sequences are intrinsically prone to spurious transcription initiation events. Nonetheless, H-NS-silenced regions remain exposed to invading transcripts that either escape repression or originate from neighboring DNA. Results presented here, support the proposal that NusG, a conserved transcription elongation factor, plays a key role in ensuring that elongation complexes come to a stop when running into a patch of oligomerized H-NS. NusG performs this function by stimulating the activity of transcription termination factor Rho. We show that alteration of this mechanism in *Salmonella* has profound physiological consequences, most likely because unstopped transcripts invade H-NS-silenced pathogenicity islands and trigger a feed-forward regulatory cascade leading to the gratuitous activation of these islands and other co-regulated loci.

## Introduction

Horizontal gene transfer has shaped the evolution of bacterial species and has had a major impact on the emergence of bacterial pathogens [1]. The phenomenon is prominent in enteric bacteria whose genomes are punctuated by sections of horizontally acquired DNA islands and islets. Acquisition of these loci allowed bacteria to gain new properties, including the ability to colonize multicellular hosts [2, 3]. Reflecting its xenogeneic nature, laterally acquired DNA has an atypical base composition, namely a higher A/T content relative to the rest of the genome [4, 5]. While providing a selective advantage in some specific environments, *e*.*g*., during host infection, expression of foreign DNA has deleterious effects on bacterial growth and can even be toxic under some conditions. Toxicity can result from various causes: *i*) the expression of lethal phage remnant genes [6]; *ii*) the energetic burden of synthesizing large macromolecular complexes (*e*.*g*., type III secretions systems) when they are not needed [7]; *iii*) the high-level sense and antisense transcription that originates from a multitude of spurious promoter-like sequences associated with A/T-rich DNA [8, 9]. Hence, *Salmonella* and related bacteria have evolved mechanisms that prevent transcription of laterally acquired genes, except when the functions of these genes are specifically needed. The main mechanism uses Histone-like nucleoid structuring protein H-NS. A small, 15.5 Kd protein, H-NS binds A/T rich DNA starting from high affinity nucleation sites and oligomerizing across adjacent sequences to form nucleoprotein filaments and bridged DNA structures [10–12]. By covering extended regions of DNA, H-NS silences multiple promoters including genuine promoters as well as spurious intragenic promoter-like sequences [13].

A second mechanism for silencing foreign DNA relies on the transcription termination activity of Rho factor. Rho is a hexameric RNA translocase that binds to nascent RNA and fueled by ATP hydrolysis, uses mechanical force to destabilize the transcription elongation complex and cause its dissociation from the DNA template [14, 15]. Rho binding sites, named Rut (Rho utilization) sites, span 50 to 100 RNA nucleotides (nt) and although lacking a consensus motif, share common sequence features, notably an excess of C over G residues (C>G skew) and the presence of UC or CC dinucleotides at 8 to 12 nt intervals to allow concomitant binding of adjacent Rho subunits [16–18]. When located in protein-coding regions of mRNAs, Rut sites are prevented from binding Rho by translating ribosomes. Hence, in these regions, Rho-dependent termination can only occur under conditions that uncouple translation from transcription [19, 20]. This notion was initially invoked to explain the upregulation of prophage genes and other horizontally acquired loci in *Escherichia coli* cells exposed to the Rho inhibiting drug bicyclomycin (BCM). It was argued that suboptimal codon usage in xenogeneic mRNA may slow-down translation thus activating cryptic Rut sites [6]. Furthermore, insertion of foreign DNA in chromosomal loci is expected to produce untranslated readthrough transcripts likely to be terminated by Rho at the first encountered Rut site [21]. Further analysis of the sites of BCM-induced readthrough provided an alternative non-mutually exclusive explanation for the Rho involvement. Landick and coworkers found that Rho terminator sites often colocalize with H-NS bound regions, suggesting that H-NS can aid Rho to terminate transcription [22]. The idea was consistent with genetic evidence showing that H-NS and related proteins can influence *rho* mutant phenotypes [23] and it has been since corroborated by additional evidence [24]. Overall, the picture emerging from this work is that certain H-NS-bound structures, particularly bridged filaments, induce pausing and backtracking of RNA polymerase and this in turns leads to Rho recruitment and termination [25, 26]. The question that remains open is how to reconcile Rho binding preference for C-rich sequences with its recruitment on A/U-rich substrate. A tentative answer to this question might be found in the participation of an additional factor, the NusG protein.

NusG is a 20 Kd protein conserved in all three domains of life, which associates with RNA polymerase after promoter clearance and affects transcription elongation in complex, contrasting ways [27]. On one hand, NusG increases RNA polymerase elongation rates and represses backtracking in vitro [28, 29]; on the other hand, NusG can promote transcription termination by stimulating the activity of Rho factor [30–32]. NusG is composed of two domains separated by a flexible linker. The N-terminal domain binds RNA polymerase while the C-terminal domain can bind either Rho or ribosomal protein S10 (NusE) [33–36]. Switching between the two C-terminus-binding partners is thought to determine whether transcription remains coupled to translation or is prematurely terminated [33, 37]. In *E*. *coli*, NusG participates in the silencing of horizontally acquired DNA, including the *kil* gene of the *rac* prophage whose toxicity renders NusG essential in this bacterium [6]. Stimulation of Rho activity by NusG is required for termination at sites with a suboptimal Rut sequence (moderate C>G skew), whereas termination is NusG-independent at sites where C>G skew is pronounced [22]. Somewhat counterintuitively in light of these findings, the stimulatory effect of NusG does not involve increasing Rho affinity for the Rut site. Rather, NusG accelerates the conformational transition–conversion from an open to a close conformation of the hexameric ring–that enables Rho to entrap the RNA and begin acting as a translocase. In doing so, NusG overrides the limitation of a weak primary interaction, allowing Rho to function on suboptimal substrates [34, 38, 39].

In this study, we combined genetic and transcriptomic approaches to assess the effects of NusG depletion on gene expression patterns in *Salmonella*. We found pathogenicity islands and other H-NS-silenced regions to be most dramatically affected, showing vast transcriptional increases as a result of NusG depletion. The same loci were upregulated in a strain carrying a mutation in the *rho* gene, K130Q, which changes a residue at the interface of the Rho subunits possibly affecting the conformational switch that accompanies Rho activity. In sharp contrast, Rho mutation Y80C, which impairs Rut site recognition, either had no effect or further tightened the repression of H-NS-silenced loci. We interpret these findings as evidence that Rho-dependent termination in H-NS-bound regions is absolutely dependent on NusG but has a relaxed Rut site binding requirement.

## Results

### Activation of H-NS-silenced genes upon NusG depletion

**Fig 1:**
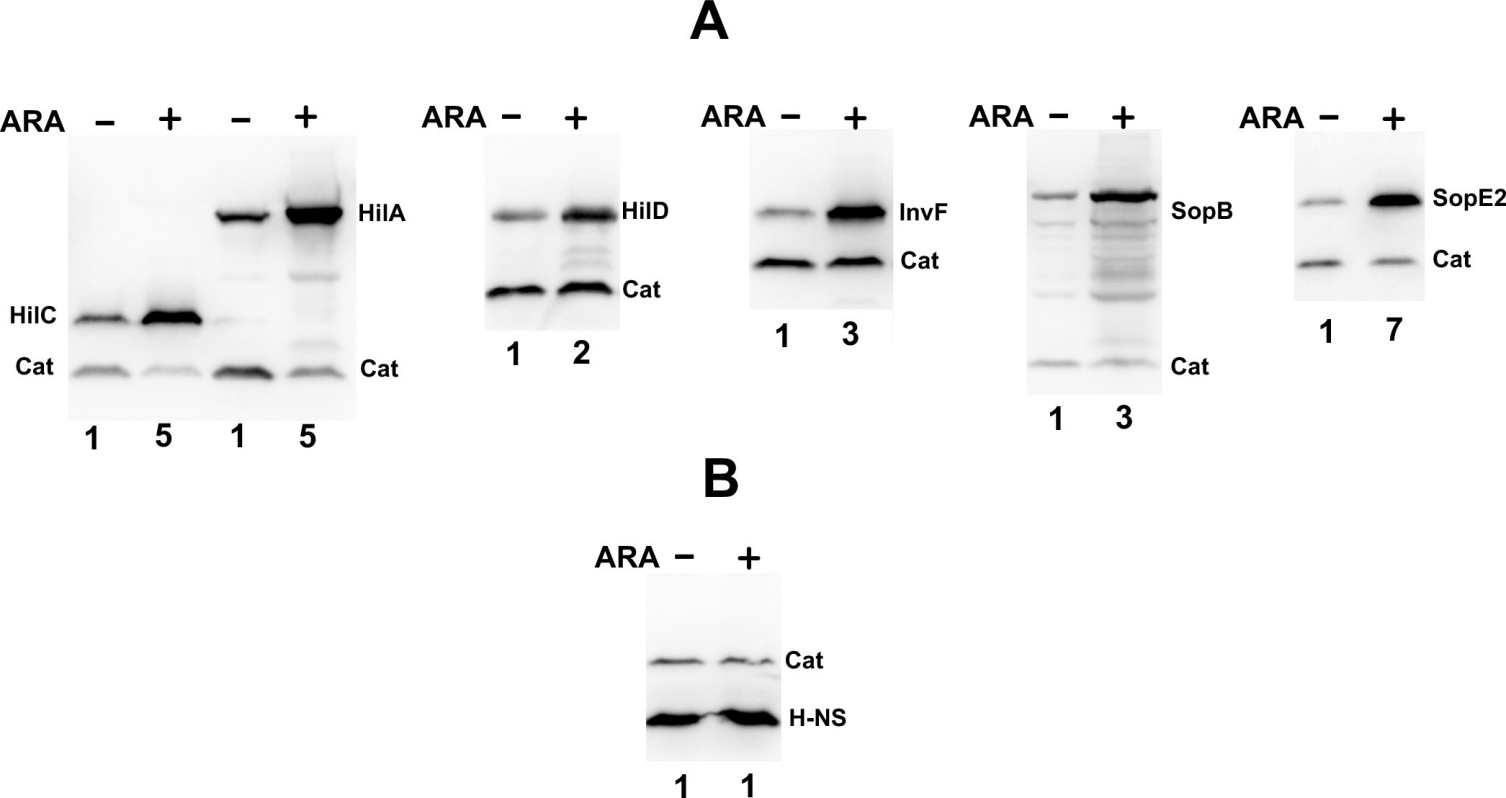
**Effect of NusG depletion on the expression of SPI-encoded proteins (A) and H-NS (B).** Strains carrying a 3xFLAG end-terminal fusion to a SPI gene, a 1xFLAG or 3xFLAG fusion to the *cat* gene (as loading control) and an arabinose inducible variant of the *nusG* gene were grown in the absence or in the presence of 0.1% arabinose to early stationary phase and processed for Western blot analysis. Values below the blots represent the fold change in the intensity of the band from the SPI-encoded protein, quantified by densitometric scanning using ImageJ, normalized to the intensity of the Cat signal for each lane. More details on the loading controls and on the cell harvesting procedure can be found in the Western blot section of Materials and methods.

A NusG-depletable strain was constructed by placing a copy of the *nusG* gene under the control of an arabinose-inducible phage repressor in the *Salmonella* chromosome, followed by the removal the endogenous *nusG* gene (S1A Fig). Ability of arabinose (ARA) to repress *nusG* expression was confirmed by Western blot analysis, which showed the levels of epitope-tagged NusG to undergo a more than 10-fold reduction upon ARA treatment (S1B Fig). To identify genes sensitive to changes in NusG level, the NusG-depletable strain was used as recipient in a transposition experiment with a *lacZY*-based transposon that generates translational *lacZ* fusions by inserting randomly in the bacterial chromosome. Approximately 50,000 transposon insertion mutants were screened for differential *lacZ* expression on indicator plates with and without arabinose. A number of clones forming white colonies (Lac^-^) on the medium without arabinose and pink, red, or deep red colonies (Lac^+^) in the arabinose-supplemented medium were identified. These clones were thus predicted to contain *lacZY* fusions to genes upregulated as a result of NusG depletion. 100 clones, exhibiting the most dramatic differential phenotypes were pooled and the chromosomal DNA mix was sequenced on an Illumina platform targeting directly the *lacZY* insertion junction. A total of 40 independent *lacZY* gene fusions were identified (S1 Table). Strikingly, 36 out of the 40 fusions were in horizontally acquired genes, mostly found in SPIs and coregulated loci. In parallel with the above analysis, a group of 10 randomly chosen clones from the starting 100 were characterized individually, determining the *lacZY* location by inverse PCR and conventional sequencing. This allowed identifying strains carrying *lacZ* fusions to *invB* (SPI-1), *sseE* (SPI-2), *sseC* (SPI-2; two independent fusions), *siiE* (SPI-4), *pipB* and *sopB* (SPI-5), *sopE2* and *sseL* (separate unnamed islands) and *leuO* (core genome). Measurement of ß-galactosidase activity confirmed NusG depletion to cause a sharp increase (ranging between 10 and 25-fold) in the expression of all 10 fusions (S2 Fig). These genes, like most of those listed in S1 Table, are known to be silenced directly or indirectly by H-NS when *Salmonella* grows in the laboratory [4, 5]. These genes are normally activated during *Salmonella* infection of a host as a result of a regulatory cascade involving a relay of multiple activator proteins including SPI-1-encoded HilA, HilC, HilD and InvF [40–43]. To assess the effects of NusG depletion on the expression of these regulators, we constructed chromosomal FLAG fusions to each of the proteins and used the strains obtained to measure protein expression levels by Western blotting before and after ARA treatment. The results of this work, which also included two of the genes analyzed as *lacZ* fusions (*invB* and *sopE2*) confirmed that all of these genes are upregulated in ARA-treated cells (Fig 1A). At the same time, no notable changes were observed in the levels of epitope-tagged H-NS (Fig 1B) ruling out possibility that NusG could act by affecting the expression of the *hns* gene. Overall, the data indicate that NusG participation in the silencing of SPI-encoded regulators is critically important to prevent gratuitous activation of *Salmonella* virulence genes.

### NusG acts in concert with Rho at most but not all sites

**Fig 2:**
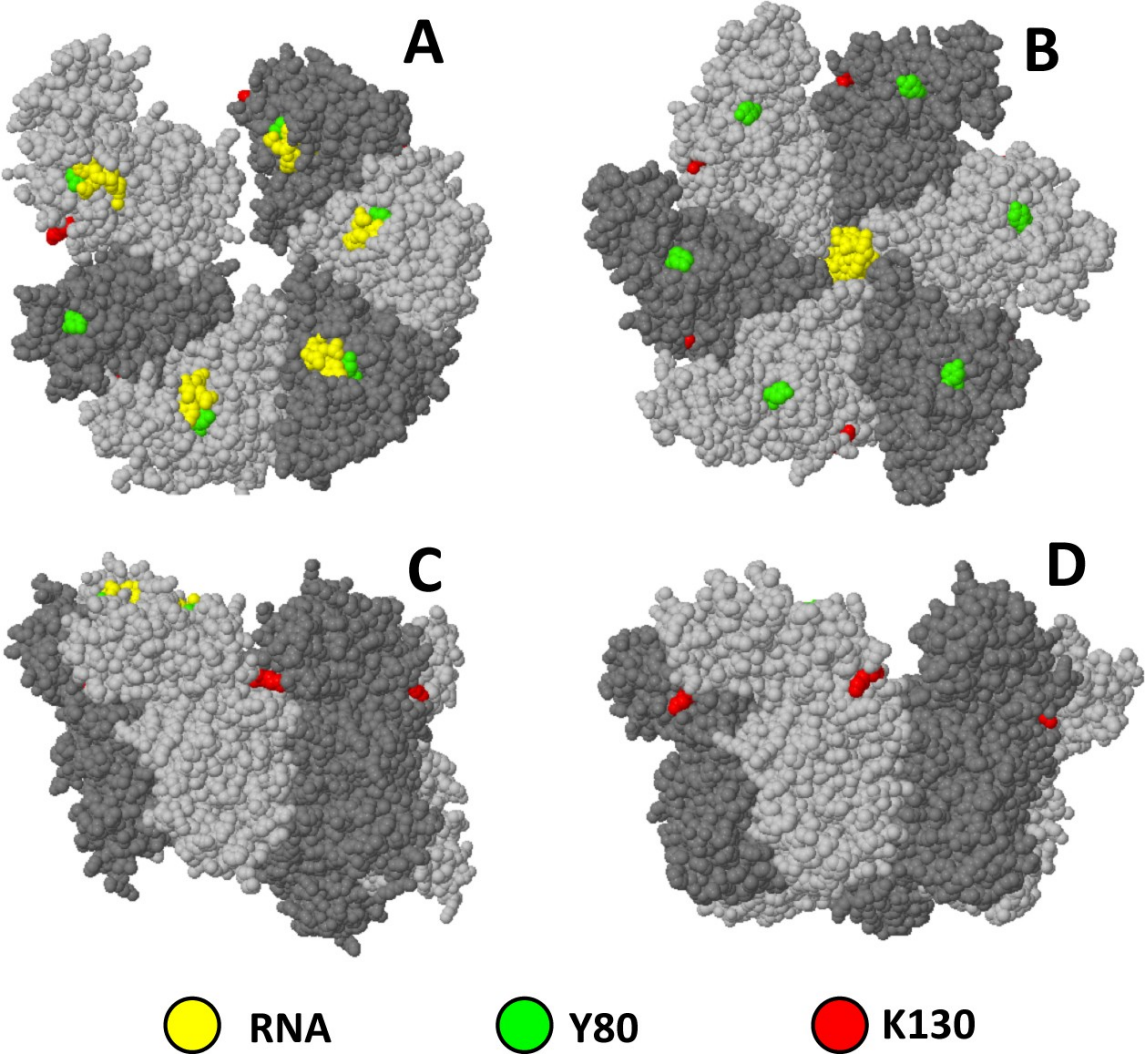
Spacefill representations of Rho protein 3D structures showing the positions of the amino acid residues changed in mutants Y80C and K130Q. Structures correspond the open and closed configurations of the Rho hexamer (*left* and *right*, refs [75] and [76], respectively) visualized with Jmol (an open-source Java viewer for chemical structures in 3D. http://www.jmol.org/). (A) and (B) *Top view*; (C) and (D) *Side view*.

**Fig 3:**
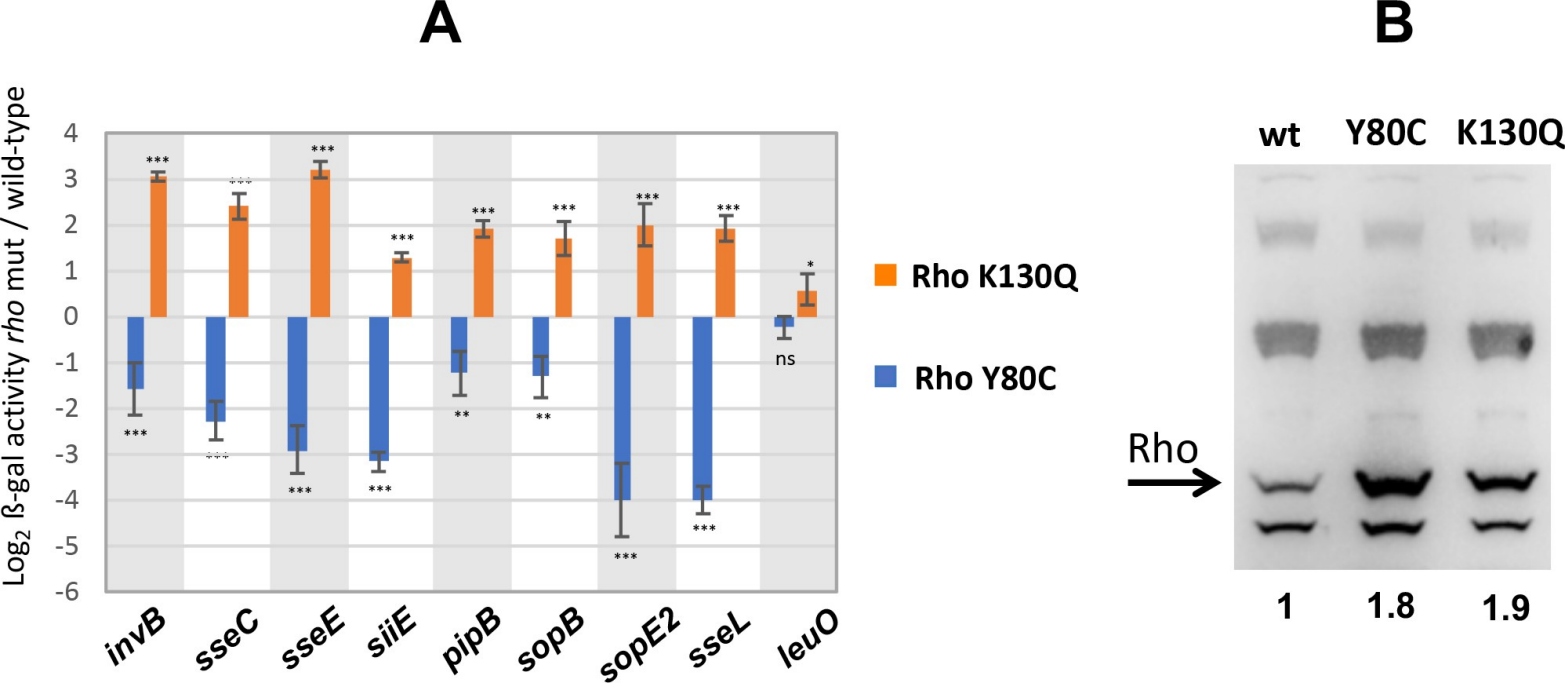
**Effects of Rho mutations on the expression of *lacZ* fusions to NusG-regulated genes (A) and on Rho protein accumulation (B).** (A) Effects are expressed as log_2_ of the ratio between the value of ß-galactosidase activity measured in the mutant versus that measured in wild-type (at least two independent assays, each with two biological replicates). Statistical significance was calculated using unpaired two-tailed Student’s T test (***, P < 0.001; **, P < 0.01; *, P < 0.05; ns, P > 0.05). Standard deviations of ratios were calculated by the formula STDV ratio = ß-gal^mut^/ß-gal^wt^ x SQRT[(STDV^mut^/ß-gal^mut^)^2^ + (STDV^wt^/ßgal^wt^)^2^]. (B) Western blot analysis of strains carrying wild-type and mutant *rho* alleles using anti-Rho polyclonal antibodies. Values below the blot represent the fold change in the intensity of the Rho band (quantified with ImageJ) normalized to the non-specific signal immediately below the Rho band.

Next, we examined the effects of Rho mutations on the gene fusions above. Two Rho alleles were used: Y80C, which lies within the primary RNA binding site affecting RNA binding [44] and termination [19, 45] and K130Q which replaces a positively charged residue by a polar residue at the interface of the Rho subunits (Fig 2). K130Q was originally isolated in a genetic selection for mutations causing increased readthrough at a weak intragenic Rho terminator dependent on NusG for activity *in vitro* and *in vivo* [19]. In the Rho crystal structure, K130 is in close proximity to the adjacent subunit when the protein is in the open ring configuration but moves farther apart in the closed conformation (Fig 2). Although the affected position lies outside the domain thought to directly interact with NusG [34], the K to Q change could still cause the structure to be more refractory to the NusG-stimulated step. K130Q was found to strongly upregulate all the fusions tested except for *leuO*. In contrast, Y80C caused the fusions to be more tightly repressed than in wild-type (Fig 3A). The latter findings were totally unexpected as in all our previous studies with the Y80C allele, we had found the mutation to increase, not decrease, the expression of genes positioned downstream from Rut sites [19, 45]. Apparently, some feature of H-NS-elicited termination might partially relieve the requirement for Rut-site binding by Rho. The actual enhancement of H-NS silencing in the presence of Rho Y80C could be explained by the increase in Rho protein levels that results from the loss of negative autogenous regulation in cells with impaired Rho activity [46] (Fig 3B).

To further assess the functional link between Rho, NusG and H-NS, and hoping to gather insight on the peculiar phenotype of the Rho Y80C mutant, we performed epistasis analysis. This involved transferring each of the two *rho* mutations in the NusG-repressible background on the one hand, and, on the other hand, combining each of them with an *hns* mutation. We used *hns* allele 123fs. This spontaneous mutant, isolated in our laboratory, carries a 20 bp deletion 24 bp away from end of the coding sequence. The resulting -1 frameshift (at codon position 123) is predicted to yield a protein of exactly the same length as wt H-NS but carrying a patch of 15 amino acid substitutions at the C-terminus. Although causing a marked defect in gene silencing (see below), *hns-123*fs impairs growth only slightly, thus limiting the risk of accumulating second-site suppressors. Results from four representative *lac* fusions showed that mutation *rho* K130Q no longer affects the expression of these fusions when NusG is depleted or *hns-123*fs is present (S3 Fig), indicating that Rho, NusG and H-NS are acting in the same pathway. In contrast, *rho* Y80C maintained its downregulation effect, albeit within a higher range of expression (S3 Fig). Note, however, that the effect is significantly attenuated in the *hns-123*fs background (*e*.*g*., from 17-fold to 1.9-fold in *sseL* and from 16-fold to 3-fold in *sopE2*; S3 Fig), again suggesting that the negative effect is specific to H-NS-elicited termination.

### NusG prevents activation of H-NS-silenced genes from external promoters

**Fig 4:**
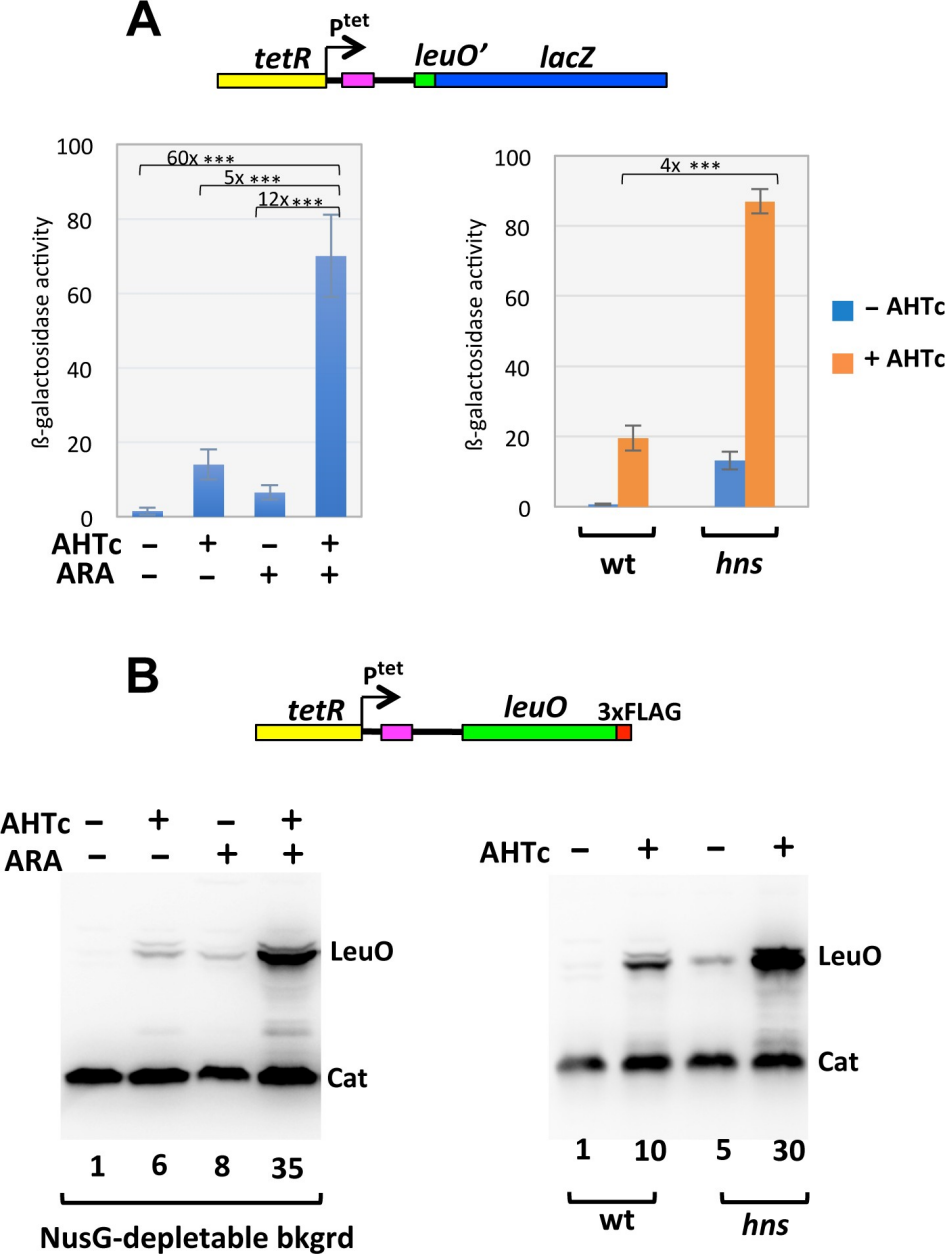
Relief of *leuO* repression by invading transcription. A DNA segment encompassing the *tetR* repressor gene and the *tetA* promoter (P^tet^) is placed upstream of the H-NS-binding site (purple shading), itself upstream of the *leuO* gene, in two separate contexts: a *leuO-lacZ* fusion (A) and a 3xFLAG-tagged version of *leuO* (B). The effects of transcription from P^tet^ (± AHTc) are analyzed in the presence or absence of NusG (- ARA and + ARA, respectively) (*left*) or in the presence of *hns* mutation *hns*-123fs (*right*). (A) Quantification of *leuO-lacZ* expression. Early stationary cultures were processed for the determination of ß-galactosidase activity as described in Materials and methods. The assays were repeated twice, each time using two independent cultures. Statistical significance was calculated by the Student’s T test (two-tailed, unpaired; ***, P < 0.001). (B) Western blot detection and quantification of LeuO-3xFLAG. Values below the blots represent the fold change in band intensity (quantified with ImageJ), relative to the untreated sample (leftmost lane), normalized to the Cat band for each lane.

The *Salmonella leuO* gene is silent under most growth conditions [47, 48] as a result of H-NS binding in the *leuO* promoter region [49, 50]. To interpret the ARA-induced upregulation of *leuO-lacZ*, one might envision that transcription originating outside the H-NS-bound region, if not stopped, can disrupt the nucleoprotein complex and activate *leuO* transcription. To test this possibility, and at the same time analyze the NusG involvement in a system where the H-NS nucleation site is well defined, we placed a *tetR*-P^tet^ cassette 57 bp upstream to the H-NS nucleation region (S4 Fig) and assessed the extent to which activating transcription of P^tet^ with Anhydrotetracycline (AHTc) affected *leuO* expression. This analysis, using the NusG depletable strain with either the *leuO-lacZ* fusion or a *leuO*-3xFLAG fusion as reporters, showed that, separately, AHTc and ARA activate *leuO* expression only slightly, but they act synergistically when combined (Fig 4, left). That is, full *leuO* expression can only occur under conditions in which transcription from P^tet^ takes place in the absence of NusG. In contrast, NusG depletion is not required to observe a significant AHTc-dependent increase of *leuO* expression in the *hfq*-123fs mutant (Fig 4, right). Taken together, the data in Fig 4 support the idea that NusG stimulates transcription termination when the elongation complex runs into a stretch of H-NS-bound DNA.

The above conclusion is independently supported by genetic data. In a strain carrying just the P^tet^-*leuO*-*lacZ* fusion, poor expression of *lacZ* causes the strain to be Lac^-^, that is, unable to grow on minimal plates with lactose as sole carbon source and supplemented with AHTc. This feature made it possible to positively select spontaneous AHTc-dependent Lac^+^ mutants. Initial characterization of four of these Lac^+^ clones showed them to form colonies smaller than the parental strain even when streaked on LB plates. This observation, combined with the slightly mucoid appearance of the mutant colonies, suggested that they might be *hns* mutants. This was confirmed by PCR amplification and sequence analysis of the *hns* gene from the mutant strains. Two mutations were found to affect H-NS N-terminal oligomerization domain (NTD; alleles E28K and ΔA46); the other two the C-terminal DNA binding domain (CTD; alleles W109R and G113D). Reasoning that the same Lac^+^ selection could be used to isolate NusG mutants, we PCR-amplified the *nusG* gene, together with a linked *cat* marker, under error-prone conditions, and introduced the mutagenized fragment into the chromosome of the strain carrying the P^tet^-*leuO*-*lacZ* fusion by DNA recombineering (selecting chloramphenicol-resistance; CamR). Replica plating the CamR colonies onto minimal lactose plates supplemented with AHTc, allowed identifying four AHTc-dependent Lac^+^ clones. Sequence analysis of the *nusG* region from these isolates confirmed the presence of *nusG* mutations. All of the changes affected N-terminal portion of the protein. Two clones carried the same allele, R8G, the remain two carried W9G and M73K. The absence of mutations in the C-terminal Rho-binding domain was tentatively consistent with the small effects of Rho mutations on the expression of the *leuO-lacZ* fusion (Fig 3A). To further address this point, we tested NusG CTD mutations isolated with a similar method in a previous study [19]. Alleles 174fs, V162D and F141S did not restore the Lac^+^ phenotype and, similarly to the Rho mutations, had smaller or no effects on *leuO-lacZ* expression (S5 Fig).

**Fig 5:**
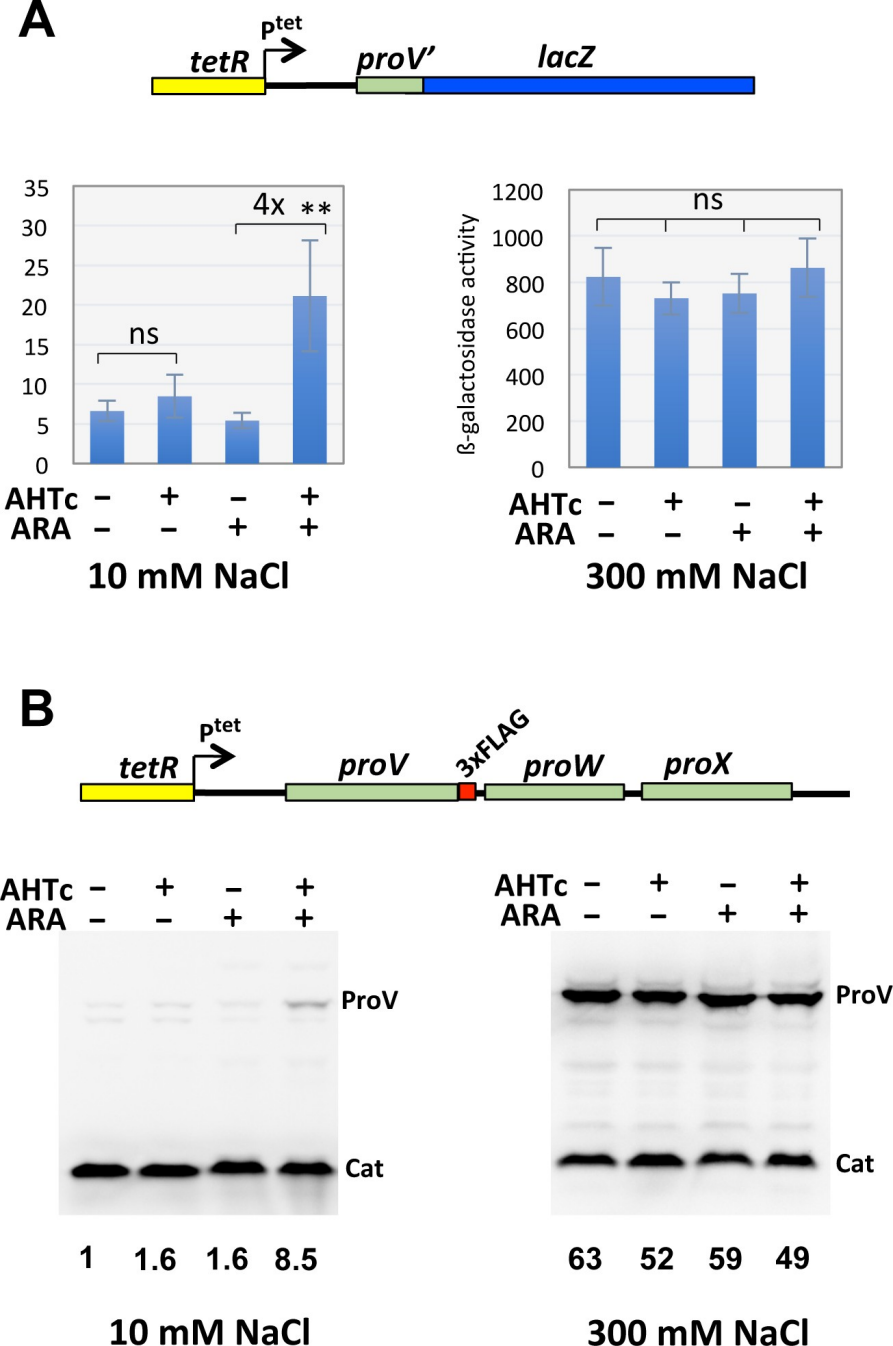
Partial relief of *proU* repression by invading transcription. The *tetR*-P^tet^ cassette was inserted 352 bp upstream from the beginning of the *proV* orf and the resulting construct moved into strains carrying either al transcriptional *lacZ* fusion placed 289 bp downstream from the beginning of the *proV* orf (A) or a C-terminal 3xFLAG fusion to *proV* (B). The effects of transcription from P^tet^ (± AHTc) are analyzed in the presence or absence of NusG (- ARA and + ARA, respectively) on early stationary cultures grown in LB medium supplemented with either 10 mM NaCl or 300 mM NaCl (high osmolarity). (A) Quantification of *proV-lacZ* expression. Cultures were processed for the determination of ß-galactosidase activity as described in Materials and methods. The assays were repeated twice, each time using two independent cultures. Statistical significance was calculated by the Student’s T test (two-tailed, unpaired; **, P < 0.01). (B) Western blot detection and quantification of ProV-3xFLAG. Values below the blots represent the fold change in band intensity relative to the untreated low osmolarity sample (leftmost lane of left blot), normalized to the Cat band for each lane. Band intensity was quantified with ImageJ.

The *proU* operon, encoding the components of an osmoprotectant transport system, constitutes another locus where H-NS binding patterns has been extensively studied [51] and references therein. At low osmolarity, H-NS binding to two sequence elements, one in the *proU* promoter region, the other inside the first structural gene, *proV*, strongly silences *proU* transcription. As a further test for NusG participation in H-NS-mediated silencing, we measured *proV* expression in strains carrying the *tetR*-P^tet^ cassette upstream of the *proU* operon and either a transcriptional *lacZ* fusion immediately downstream from the internal H-NS binding site or a chromosomal *proV*-3xFLAG fusion. Like in the *leuO* experiments, the analysis was performed in the presence or absence of AHTc and/or ARA. Results showed that P^tet^ activation produces a 4- to 8-fold increase in *proV* expression at low osmolarity but only on condition that AHTc is added together with ARA, whereas no significant change was observed with AHTc alone (Fig 5A). Although the overall induction ratio remains much lower than that attained at high osmolarity (which, incidentally, is not significantly affected by AHTc ± ARA; Fig 5B) these data further indicate that relief of H-NS-mediated silencing by invading transcription can only occur if NusG is removed from the elongation complex.

Prior to this work, Rangarajan and Schnetz, reported that directing transcription toward the *proU* operon, from a location close to that of P^tet^ in our study, activated *proU* transcription [52]. This work was carried out in an *E*. *coli* strain with normal NusG levels, from which one would infer that NusG plays no role in that system. However, we notice that some of the key experiments in this report were performed in the presence of phage lambda anti-terminator protein N, that is, under conditions that would prevent NusG-stimulated termination [52]. Furthermore, the use of quantitative PCR for transcript detection and quantification, as done by the above authors, could have revealed differences undetectable by the biological approach used in our study.

### Whole transcriptome analysis

**Fig 6:**
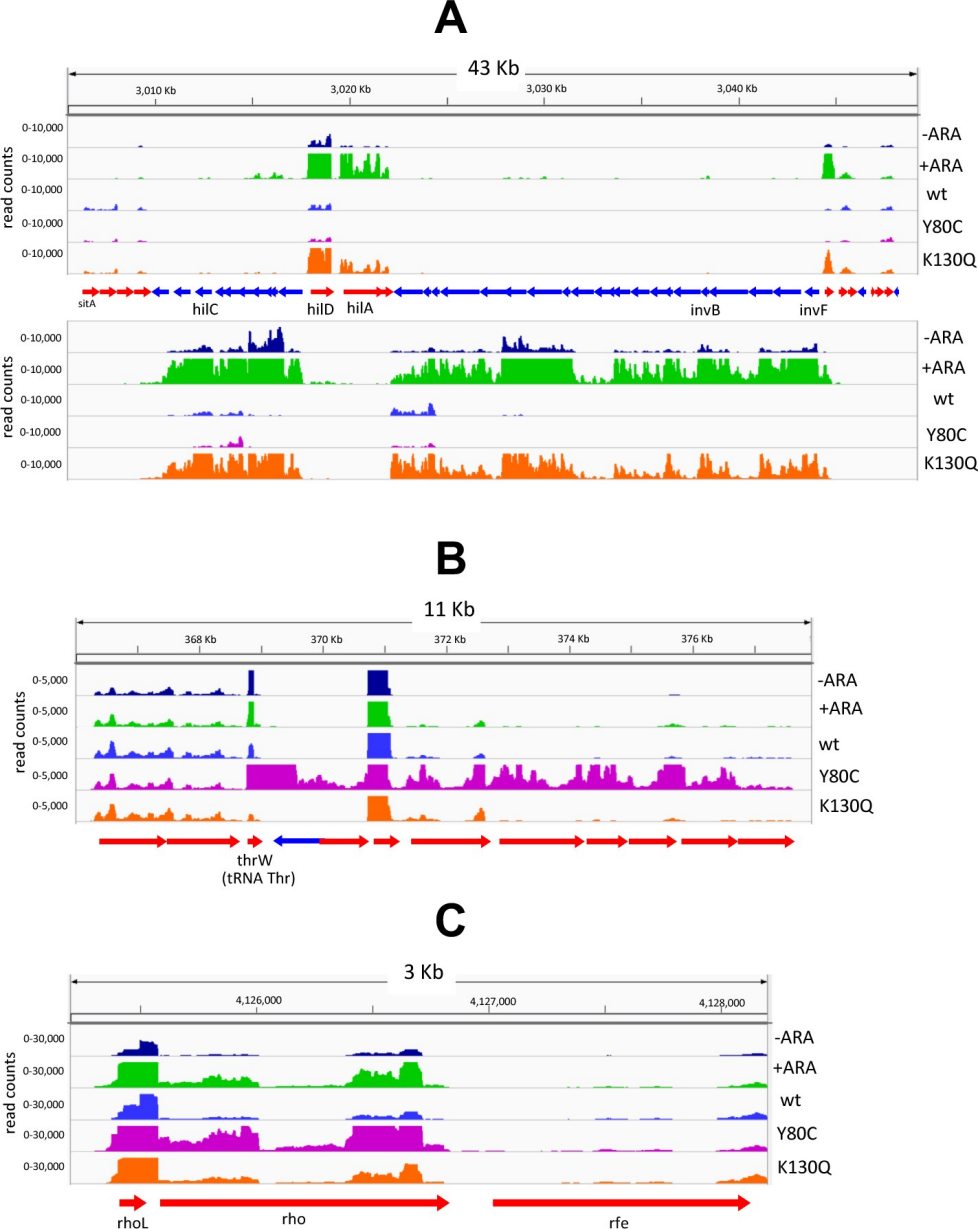
Effects of NusG depletion or Rho mutations on RNA profiles in representative regions. Profiles above arrows correspond to sense transcription of right-oriented genes (red arrows); profiles below arrows correspond to sense transcription of left-oriented genes (blue arrows). (A) SPI-1; (B) Chromosomal region downstream from the *thrW* gene; (C) *rho* gene region. Data are visualized with Integrative Genome Viewer (IGV) [77].

**Fig 7:**
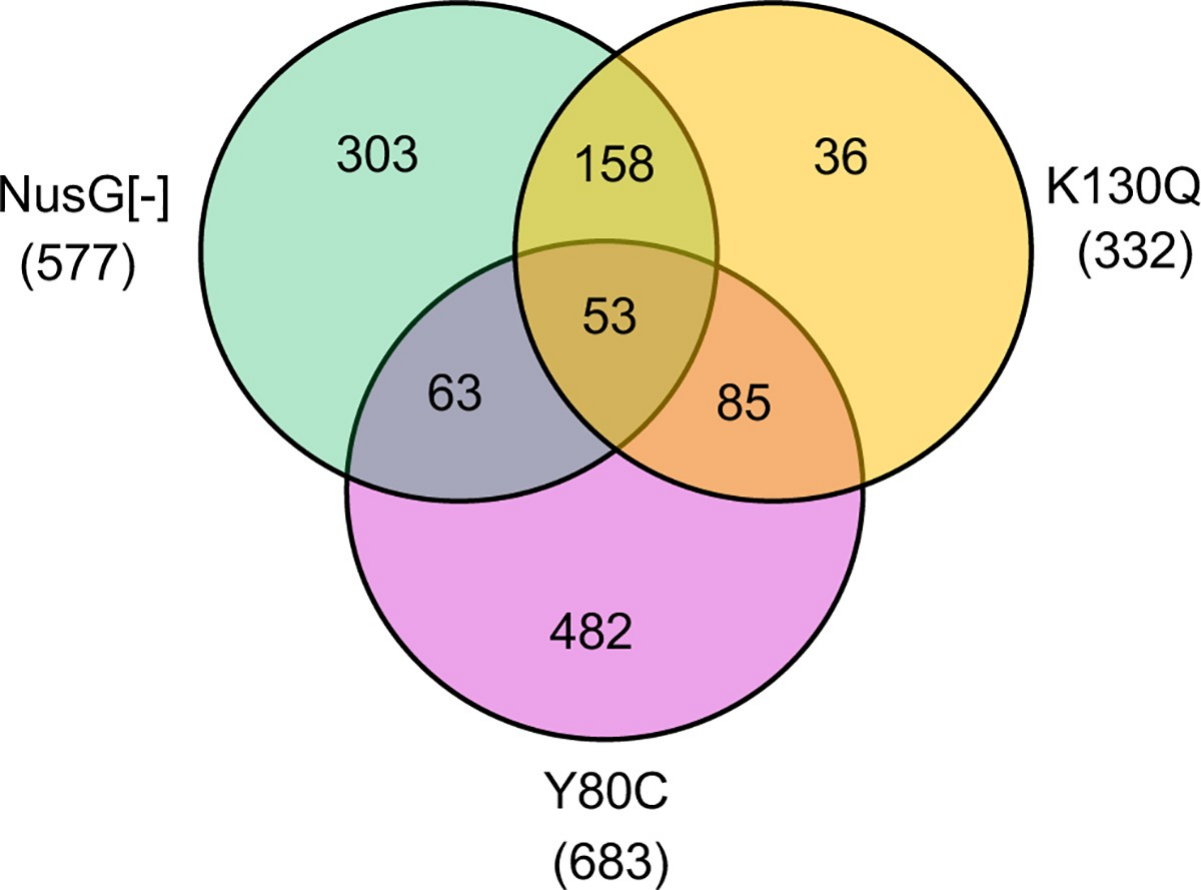
Venn diagram showing the distribution of genes affected by NusG depletion and/or Rho mutations. Only genes whose transcript levels increase 2-fold or more (log2FoldChange versus wt ≥ 1; padJ ≤ 0.05) under at least one of three conditions applied in this study (ARA treatment; Rho mutants Y80C and K130Q) were considered. Circle overlap areas denote genes responding to more than one condition. The gene names are listed in S2 Table. Note that for some genes, group assignment is critically dependent on the log2FoldChange and padJ cutoff values. For example, by setting log2FoldChange to ≥ 0.8 and padJ to ≤ 0.01, the number of genes responding to K130Q and not to the other conditions drops to 9.

To extend the above analysis, the NusG-depletable strain and the two Rho mutants were subjected to high throughput RNA sequencing (RNA-seq). The transcriptional profiles are directly accessible for viewing using the intuitive JBrowse interface [53] at (http://salmonella.cnrs-orleans.fr/NusG-Rho/). This analysis confirmed that the most dramatic differences occur in pathogenicity islands and other H-NS-silenced loci where transcript levels undergo a several-fold increase upon NusG depletion (see examples in Fig 6A and S6 Fig). The RNA profiles of the Rho K130Q mutant mimic those of NusG-depleted cells at several positions. About 65% of all genes upregulated more than 2-fold in the Rho K130Q strain are upregulated to the same or higher extent during NusG depletion (Fig 7; S2 Table). The RNA increase is not confined to the sense strand but it is also significant in the antisense strand (*e*.*g*., see *siiE*, *sopB* and *pagC* in S6A Fig, S6B Fig and S6C Fig, respectively), supporting the idea that NusG and Rho act in concert to suppress pervasive sense and antisense transcription in H-NS-bound regions of the genome [22]. Note that in many regions, the basal RNA levels of the NusG-repressible strain (the [–]ARA samples) are somewhat higher than the corresponding RNA levels in wild-type, for example, in left-oriented genes in Fig 6A. We ascribe the difference to the fact that in the NusG-repressible strain, the *nusG* gene is transcribed from a prophage promoter, which may be slightly weaker than the natural *nusG* promoter in the wild-type strain. As a result, NusG levels could be already somewhat lower prior to ARA addition. As anticipated from the analysis of the *lacZ* fusions, the Rho Y80C mutant displays a quite different transcriptomic profile. Rho Y80C has either no effect or downregulates many of the genes upregulated by the NusG depletion (S2 Table). Conversely, the majority of genes whose expression increases more than two-fold in the Y80C strain (about 75%; Fig 7) are not significantly affected by NusG depletion or the K130Q mutation. Dramatic examples of this pattern can be seen on the 3’ side of tRNA loci, where failure of Rho Y80C to terminate transcription results in massive readthrough transcription of several downstream genes (Fig 6B and S7 Fig). The absence of any significant alteration in the RNA profiles of NusG-depleted cells or K130Q mutant cells at this position is striking and confirms that, in the presence of a strong Rut site, Rho acts independently of NusG and is insensitive to changes that affect the NusG-dependent step(s) [22]. Next to these differences, there are several instances where the effects of Rho Y80C closely parallel those of NusG depletion (S2 Table): one such example is the *rho* gene itself (Fig 6C). Interestingly, from the higher levels of *rho* mRNA in the ARA-treated culture, one would infer that NusG participates in the negative autogenous regulation, something that to the best of our knowledge was not known prior to this study. Also interesting to notice is that at the RNA level, Y80C appears to affect *rho* regulation more than K130Q. Finally, there is a class of loci that reproduce the *leuO* pattern, in that they are strongly activated in NusG-depleted cells while showing little or no change in the presence of either of the two Rho mutations. A relevant member of this class is the *slyA* gene (S2 Table and S8 Fig), which encodes a factor that can antagonize H-NS at some sites [54, 55]. The complete lists of genes whose transcription levels change significantly under any of the conditions analyzed in this study are shown in S5–S**7** Tables. Overall, the results from the transcriptomic analysis confirm and extend the conclusions drawn from the genetic analysis.

## Discussion

This study shows that transcription elongation factor NusG is critically required for gene silencing by nucleoid structuring protein H-NS in *Salmonella*. NusG activity appears to be needed to prevent spurious transcription originating in the proximity of H-NS-silenced regions from invading these regions and disrupting H-NS nucleoprotein complexes. Failure to block this transcription, in NusG-depleted cells, results in the upregulation of *Salmonella* pathogenicity islands and a variety of other loci. SPIs are normally maintained repressed by H-NS and activated via a feed-forward regulatory cascade involving autogenously controlled activator proteins encoded within H-NS-silenced domains [40–43]. Disruption of the silencing structure by invading transcription could transiently free the promoters of the activator genes [52]. The self-activating nature of the regulatory cascade is likely to account for the magnitude of the changes observed here and for our ability to detect these changes at both the RNA and protein levels. This was seldom the case in previous studies. For example, out of 101 proteins whose levels were found increased more than two-fold in *E*. *coli* cells exposed to Rho inhibitor bicyclomycin, only one encoded by a horizontally acquired gene, RecE, was also upregulated at the RNA level [6]. This lack of correlation between RNA and protein profiles remains unexplained.

Overall, the main findings described here are consistent with the idea that transcribing RNA polymerases slow-down or pause when running into H-NS-bound DNA and this opens a time window during which NusG recruits Rho factor and promotes transcription termination [25, 26]. The Rho involvement in the silencing of horizontally acquired DNA is supported by a large body of evidence in *E*. *coli* [6, 21, 22] and in some gram-positive bacteria [56–58]. While some of these studies recognized NusG participation in these effects [6, 22], the question as to whether NusG were actually required for Rho action at H-NS-silenced sites had not been previously addressed. Finding that Rho mutation K130Q is completely epistatic to NusG depletion constitutes strong genetic evidence that Rho and NusG act in the same pathway.

Remaining unclear is the response to Rho Y80C. This mutation lies within the primary RNA binding sites of Rho (Fig 2) and was shown to impair Rut site binding [44]. In vivo, Rho Y80C increases read-through transcription at sites that are normally terminated by Rho [19, 45]. So, how to explain that the mutation enhances silencing by H-NS, which implies that Y80C could actually improve termination at H-NS-bound sites? One possibility is that termination at these sites does not require, or is less dependent upon, Rut site binding. For example, one can imagine that Rho recruitment by NusG could partially or even completely bypass the Rut site-binding requirement. Previous demonstration that NusG stimulates Rho activity at suboptimal Rut sites is tentatively consistent with this idea [22, 34]. However, this model predicts that hyper-repression by Y80C should be relieved, or strongly attenuated, under NusG-depleted conditions, which was not observed in our study. The only condition where we found the Y80C effects to be significantly diminished was in the presence of an *hns* mutation. This confirms that the effects are H-NS-specific and might be interpreted to suggest that a transcriptional roadblock suppresses the Rut binding defect. Perhaps, stalling of RNA polymerase at the boundaries of H-NS-bound sites allows more time for the mutant Rho to bind to a Rut site and complete its cycle. The increase in Rho protein levels consequent to the Y80C mutation (see Results) would then account for the hyper-repression. Clearly, more work will be needed to test this model. The problem is further complicated by the observation that Y80C causes allosteric changes in other parts of the protein, affecting ATP binding and RNA release [44]. Finally, one should mention that Rho Y80C was found to suppress the cytotoxic effects of overexpressing of phage P4 antiterminator protein Psu in *E*. *coli* [59], a finding suggesting that Rho Y80C can increase termination under certain conditions. The analogy with the observations of our study is intriguing but remains unexplained.

A fraction of loci that are upregulated when NusG is depleted (*e*.*g*., *leuO*), show little or no change in expression in the presence of either of the Rho mutations analyzed here. Currently, it is unclear whether this lack of effect is ascribable to the mutant Rho proteins remaining fully proficient for termination at some sites, or if NusG can occasionally promote termination or cause transcription to abort by a Rho-independent mechanism. Finding that mutations in NusG NTD (involved in RNA polymerase binding), but not in NusG CTD (involved in Rho recruitment), relieve H-NS repression of *leuO* (S5 Fig) seems more consistent with the latter scenario. The presence of the *slyA* gene within the upregulated group (S8 Fig) raises the possibility that some of the members of the group are SlyA-activated genes. Like with *leuO*, the mechanism underlying *slyA* upregulation in NusG-depleted cells remains elusive.

Regulation of the elongation phase of transcription is important for coordination with other cellular mechanisms [60] and for preserving genome integrity [61]. Conservation of NusG and its functional homologs in all three kingdoms of life highlights the importance of this regulation. The NusG family proteins assist RNA polymerase progression and link transcription to other processes through the recruitment of accessory factors. While a detailed picture of how NusG affects RNA polymerase mechanics is beginning to emerge [62], the recruiting pathway remains ill-defined. Gaining a better understanding of this pathway will not only profit the prokaryotic field but can also help in the characterization of similar mechanisms in eukaryotic cells.

## Materials and methods

### Bacterial strains and culture conditions

Strains used in this work are derived from *Salmonella enterica* serovar Typhimurium strain MA3409, a derivative of strain LT2 [63] cured for the Gifsy-1 prophage [64]. The genotypes of the relevant strains used are listed in S3 Table. Bacteria were cultured at 37°C in liquid media or in media solidified by the addition of 1.5% Difco agar. LB broth [65] was used as complex medium. Carbon-free medium (NCE) [66], supplemented with 0.2% glycerol or 0.2% lactose was used as minimal medium. When needed, antibiotics (Sigma-Aldrich) were included in growth media at the following final concentrations: chloramphenicol, 10 μg/ml; kanamycin monosulphate, 50 μg/ml; sodium ampicillin 100 μg/ml; spectinomycin dihydrochloride, 80 μg/ml; tetracycline hydrochloride, 25 μg/ml.

### Genetic techniques

Strain construction was done by generalized transduction using the high-frequency transducing mutant of phage P22, HT 105/1 *int-201* [67] or by the λ-*red* recombineering technique implemented as in [68]. 3xFLAG epitope fusions were constructed as described [69]. DNA Oligonucleotide used as primers for PCR amplification (obtained from Sigma-Aldrich or Eurofins) are listed in S4 Table. PCR-amplified fragments to be used for recombineering were produced with high-fidelity Phusion polymerase (New England Biolabs). Constructs were verified by colony-PCR using Taq polymerase followed by DNA sequencing (performed by Eurofins-GATC Biotech). Detailed description of the steps involved in the construction of the most relevant strains used in this work can be found in Supplementary Methods.

### Tn*5*-*lac* transposition

A plasmid suitable for use as a *lacZY* transposon delivery vector was constructed at the onset of this study. This plasmid, pTn*5*-*lac*-SHP carries a DNA segment encompassing the *lacZ-lacY* genes and a kanamycin resistance cassette, the whole flanked by the 19 bp terminal repeats recognized by the Tn*5* transposase. Adjacent to the transposable segment, lies the gene encoding the hyperactive variant of Tn*5* transposase [70] under the control of a *tetR*-P^tet^ cassette. Finally, pTn*5*-*lac*-SHP carries a minimal R6K replication origin and cannot replicate in the absence of the π protein. When pTn*5*-*lac*-SHP is used to transform suitable recipient cells, kanamycin-resistant clones can only arise from Tn*5*-mediated transposition. Since the *lacZ* part of the transposon lacks transcription and translation initiation signals, *lacZ* expression can only occur if the *lacZ* sequence inserts in-frame within a protein-encoding gene. In the present study, pTn5-lac-SHP DNA was introduced into NusG-depletable strain MA12996 by electro-transformation. Transformed cells were incubated 1.5 hours in LB medium supplemented with AHTc (0.4 μg/ml) at 37°C. Cells were then plated on LB plates supplemented with Kanamycin. Approximately 50,000 Kanamycin-resistant colonies were replica-plated on MacConkey lactose indicator plates [71] with or without arabinose. A number of clones forming white colonies (Lac^-^) on the medium without arabinose and pink, red or deep red colonies (Lac^+^) in the arabinose-supplemented medium were identified.

### Identification of *lacZY* insertion sites

A. *Tn-*lac*-seq*. Genomic DNA from a pool of insertion mutants showing differential lacZ expression was prepared for deep sequencing by a modified version of the Nextera Tn-seq protocol [72]. Briefly, DNA was first tagmented using Tagment DNA enzyme 1 from the Nextera DNA Library Preparation Kit (Illumina). Next, nested PCR was carried out, first amplifying the region between the beginning of *lacZ* and one of the Nextera transposomes (primers Tn5lacZ-R1 and 2A-R) The second PCR added specific indices and Illumina end sequences needed for sequencing on the Illumina platform (primers lacZ-R2 and N712). The final PCR products were then pooled and purified using the QIAquick PCR Purification Kit (Qiagen). Sequencing was performed at Tufts University Core Facility (tucf.org) by single-end, 50 cycle, high output mode sequencing on a HiSeq2500 machine using custom sequencing primer Tn5lacZ-seq.

B. *Individual analysis of selected insertions*. In parallel with the Tn-*lac*-seq analysis, a group of 10 randomly chosen clones were characterized individually by inverse PCR as previously described [73]. Briefly, this involved *i*) genomic DNA preparation from each of the clones, *ii*) cleaving the genomic DNA with restriction endonuclease HaeII (New England Biololabs), *iii*) circularization of the resulting fragments with T4 DNA ligase (low concentration, ref. 15224041 ThermoFisher Scientific, USA) and *iv*) using the ligated mixture for PCR-amplification with divergent primers from either end of the transposon: primers ppX52-ppX53 from the left end (the *lacZ* end); primers ppAB05-ppAB06 from right end. Fragments obtained were subjected to conventional Sanger sequencing.

### Western blot analysis

Strains to be analyzed were grown from single colonies in 0.4 ml of LB (in 2 ml microcentrifuge tubes) overnight at 37°C without shaking. Overnight cultures were diluted 1:200 in 2 ml of LB (with or without the appropriate supplements) and grown with shaking at 37°C for 6 to 7 hours. 1 ml aliquots were centrifuged (2 min 12000, rcf) and pellets resuspended in 50–100 μl of H_2_O. 10 μl were withdrawn, diluted 1:100 and used to measure OD600. The values obtained were used to equalize the densities of cell suspensions, correcting differences due to treatments or genotypes. The adjusted bacterial suspensions were then mixed with one volume of 2x Laemmli buffer, lysed by boiling (5 min) and loaded onto polyacrylamide gels. From this point on, samples were processed for Western blot analysis essentially as described [69] with minor modifications (see Supplementary Methods for details). Blots were imaged on the ChemiDoc Touch imaging system (BioRad). In experiments involving the FLAG epitope, the strains analyzed typically carried a second copy of the FLAG peptide fused to a chromosomal *cat* gene to serve as gel loading control. To prevent that image saturation of the *cat* band could limit the range of analysis in the rest of the blot, whenever possible we used a *cat* control construct producing a signal of lower intensity than the protein of interest. This required replacing the 3xFLAG epitope with the more weakly detectable lxFLAG epitope. Protein bands were quantified using ImageJ freeware.

### RNA-Seq

Overnight bacterial cultures were diluted 1:200 in LB medium or in LB medium supplemented with 0.1% arabinose where appropriate and grown with shaking at 37°C to an OD_600_ = 0.7 to 0.8). Three samples were prepared from independent biological replicates for each condition or strain. Cultures (4 ml) were rapidly spun down and resuspended in 0.6 ml ice-cold REB buffer (20 mM Sodium Acetate pH 5.0, 10% sucrose). RNA was purified by sequential extraction with hot acid phenol, phenol-chloroform 1:1 mixture and chloroform. Following overnight ethanol precipitation at -20°C and centrifugation, the RNA pellet was resuspended in 12 μl of H_2_O. Subsequent steps were carried out at the Next Generation Sequencing (NGS) Core Facility of the Institute for Integrative Biology of the Cell, CNRS, Gif-sur-Yvette, France. This work is described in Supplementary Methods.

### Measurement of β-galactosidase activity

Strains to be assayed were grown from single colonies in 0.4 ml of LB (in 2 ml microcentrifuge tubes) overnight at 37°C without shaking. Overnight cultures were diluted 1:200 in 2 ml of LB (with or without the appropriate supplements) and grown with shaking at 37°C for 6 to 7 hours. At this time the OD600 values typically ranged between 2 and 3.5 depending of strain genotype and growth conditions. Bacteria were harvested from 1 ml aliquots and resuspended in 1 ml of PBS. β-galactosidase activity was assayed in toluene-permeabilized cells as described by Miller [74] and is expressed in Miller units or as percentage of the activity measured in the wild-type strain in the same experiment. Measurements were repeated twice each time using duplicate cultures originating from independent colonies.

## Supporting information

S1 Fig Arabinose-induced NusG depletion. (A) Diagram showing the main features of the NusG depletable strain. This strain (MA12996) carries the Gifsy-2 prophage repressor gene (*gtgR*) under the control of the arabinose operon promoter (P^BAD^). Gifsy-2 left operon promoter (P^G2L^, lying on the 3’ side of *gtgR*) is fused to the coding sequence of *nusG*. The native copy of the *nusG* gene as well as the entirety of Gifsy-1 and Gifsy-2 prophages are deleted. (B) Western blot analysis of a strain (MA13953) carrying 3xFLAG epitope fusions to the 3’ end of the GtgR-repressible *nusG* and to the *cat* gene (as an internal standard) grown in the absence or in the presence of 0.1% arabinose. Values below the blot represent the fold change in the intensity of the NusG band before and after the ARA treatment, normalized to the intensity of the *cat* signal in the same lane.
(TIF)

S2 Fig Upregulation of pathogenicity-related genes in NusG depleted cells. *lacZ* gene fusions to genes from pathogenicity islands or related loci were obtained following random *lacZ* transposition in a strain carrying the *nusG* gene under the control of an arabinose-inducible repressor. Strains carrying the various fusions were grown in the presence or absence of arabinose to early stationary phase (OD_600_ = 2–3.5) and assayed for ß-galactosidase activity (two separate assays, each performed on two independent cultures). Statistical significance was calculated using unpaired two-tailed Student’s T (***, P < 0.001).
(TIF)

S3 Fig Epistasis analysis of the Rho-NusG-H-NS connection. Strains carrying wild-type or mutant alleles of *rho* (*rho* Y80C or K130Q) in combination with either a wild-type or an ARA-repressible version of the *nusG* gene, or with either wild-type or mutant *hns* (*hns*-123fs), and one of four different *lacZ* fusions identified in this study, were grown to early stationary phase and assayed for ß-galactosidase activity as described in Materials and methods. Assays were performed at least twice, each time with two biological replicates. Statistical significance was calculated by the Student’s T test (unpaired two-tailed; ***, P < 0.001; **, P < 0.01; *, P < 0.05; ns, P > 0.05). Values in vertical axes represent Miller units of ß-galactosidase activity [74].
(TIF)

S4 Fig DNA sequence (sense strand) of the region between the start of transcription from P^tet^ (+1) and the beginning of *leuO*. The *leuO* promoter sequence (yellow boxes) and the H-NS binding site (purple lettering) are from refs [49] and [50], respectively.
(TIF)

S5 Fig Effects of NusG and Rho mutations on H-NS-mediated silencing of a P^tet^-*leuO-lacZ* fusion. A strain carrying P^tet^-*leuO-lacZ* is phenotypically Lac^-^ in the presence of the P^tet^ inducer (AHTc), because H-NS prevents transcription from reaching the *leuO* coding sequence. NusG NTD mutations W9G, R8G and M73K were isolated selecting Lac^+^ derivatives. NusG CTD mutations 174fs, V162D and F141S and Rho mutations Y80C and K130K were isolated previously (see main text). Strains carrying these different alleles were grown in the presence or absence of AHTc (0.4 μg/ml) to early stationary phase and assayed for ß-galactosidase activity (two independent assays, with two biological replicas each). Statistical significance of each mutant versus wild-type difference in AHTc-supplemented cultures was calculated by the Student’s T test (***, P < 0.001; **, P < 0.01; ns, P > 0.05). Results show that NusG NTD mutations are significantly more effective than NusG NTD and Rho mutations at relieving the H-NS block. Although some of the latter do cause some increase in *lac* expression, the increase is not sufficient to render the strain Lac^+^.
(TIF)

S6 Fig Effects of NusG depletion or Rho mutations on RNA profiles in pathogenicity islands. Profiles above and below arrows correspond to sense transcription of right-oriented genes (red arrows) and left-oriented genes (blue arrows), respectively. (A) SPI-4; (B) SPI-5; (C) SPI-11. Note the increase of anti-sense transcription throughout SPI-4 (A), in the *pipC*-*sopB* region of SPI-5 (B) and in the *pag*C locus (C) during NusG depletion.
(TIF)

S7 Fig Effects of NusG depletion or Rho mutations on RNA profiles downstream from representative tRNA genes or operons. Profiles above arrows correspond to sense transcription of right-oriented genes (red arrows); profiles below arrows correspond to sense transcription of left-oriented genes (blue arrows). (A) *glyU*; (B) *glyV glyX glyY;* (C) *leuV*, *leuP*, *leuQ*; (D) *serV argVα argVβ argVγ argVδ*.
(TIF)

S8 Fig Effects of NusG depletion or Rho mutations on RNA profiles in the *slyA* gene. Profiles above the red arrow correspond to *slyA* sense transcription; profiles below the arrow correspond to *slyA* anti-sense transcription.
(TIF)

S1 Table Translational *lacZ* gene fusions upregulated in NusG-depleted cells. Random *lacZY* transposon insertions mutants showing increased *lacZ* expression under NusG depletion conditions were isolated, pooled and sequenced as described in the text.
(XLSX)

S2 Table Comparing upregulated gene patterns in cells depleted for NusG or carrying a Rho mutation. The gene listed show at least a two-fold change in RNA levels under any of the indicated conditions (padJ ≤ 0.05).
(XLSX)

S3 Table *Salmonella enterica* serovar Typhimurium strains used in this work. All strains used in this work are derived from a *Salmonella enterica* serovar Typhimurium strain LT2 derivative cured for the Gifsy-1 prophage. Strains were constructed by phage P22-mediated transduction and/or λ-*red* recombineering as described in the text.
(XLSX)

S4 Table DNA oligonucleotides used in this work. Red lettering denotes annealing sequences in oligonucleotides used for λ-*red* recombineering.
(XLSX)

S5 Table Genes differentially expressed in NusG-depleted cells. Comparison of RNA levels in strain MA12996 grown in the presence or absence of 0.1% arabinose. Differential expression was computed by analyzing the RNA Seq data with DESeq2. Only genes with a padJ value ≤ 0.05 are listed.
(XLSX)

S6 Table Genes differentially expressed in Rho Y80C mutant cells. Comparison of RNA levels in strains MA13775 (*rho* wt) and MA13776 (*rho* Y80C). Differential expression was computed by analyzing the RNA Seq data with DESeq2. Only genes with a padJ value ≤ 0.05 are listed.
(XLSX)

S7 Table Genes differentially expressed in Rho K130Q mutant cells. Comparison of RNA levels in strains MA13775 (*rho* wt) and MA13778 (*rho* K130Q). Differential expression was computed by analyzing the RNA Seq data with DESeq2. Only genes with a padJ value ≤ 0.05 are listed.
(XLSX)

S8 Table Source data for Figures. Quantification of Northern blot hybridization signals in gels of Figs 1, 3B, 4B, 5B and S1 Fig (densitometric scanning using ImageJ) and ß-galactosidase activity determinations for the experiments shown in Figs 3A, 4A and 5A.
(XLSX)

S1 Methods (DOCX)

## References

[1] Ochman H, Lawrence JG, Groisman EA. Lateral gene transfer and the nature of bacterial innovation. Nature. 2000;405(6784):299–304. 10.1038/35012500 . 10830951

[2] Diard M, Hardt WD. Evolution of bacterial virulence. FEMS Microbiol Rev. 2017;41(5):679–97. 10.1093/femsre/fux023 . 28531298

[3] Groisman EA, Ochman H. Pathogenicity islands: bacterial evolution in quantum leaps. Cell. 1996;87(5):791–4. 10.1016/s0092-8674(00)81985-6 . 8945505

[4] Lucchini S, Rowley G, Goldberg MD, Hurd D, Harrison M, Hinton JC. H-NS mediates the silencing of laterally acquired genes in bacteria. PLoS Pathog. 2006;2(8):e81 10.1371/journal.ppat.0020081 . 16933988 PMC1550270

[5] Navarre WW, Porwollik S, Wang Y, McClelland M, Rosen H, Libby SJ, et al Selective silencing of foreign DNA with low GC content by the H-NS protein in Salmonella. Science. 2006;313(5784):236–8. 10.1126/science.1128794 . 16763111

[6] Cardinale CJ, Washburn RS, Tadigotla VR, Brown LM, Gottesman ME, Nudler E. Termination factor Rho and its cofactors NusA and NusG silence foreign DNA in E. coli. Science. 2008;320(5878):935–8. 10.1126/science.1152763 . 18487194 PMC4059013

[7] Sturm A, Heinemann M, Arnoldini M, Benecke A, Ackermann M, Benz M, et al The cost of virulence: retarded growth of Salmonella Typhimurium cells expressing type III secretion system 1. PLoS Pathog. 2011;7(7):e1002143 10.1371/journal.ppat.1002143 . 21829349 PMC3145796

[8] Lamberte LE, Baniulyte G, Singh SS, Stringer AM, Bonocora RP, Stracy M, et al Horizontally acquired AT-rich genes in Escherichia coli cause toxicity by sequestering RNA polymerase. Nat Microbiol. 2017;2:16249 10.1038/nmicrobiol.2016.249 . 28067866 PMC7610989

[9] Wade JT, Grainger DC. Pervasive transcription: illuminating the dark matter of bacterial transcriptomes. Nat Rev Microbiol. 2014;12(9):647–53. 10.1038/nrmicro3316 . 25069631

[10] Ali SS, Xia B, Liu J, Navarre WW. Silencing of foreign DNA in bacteria. Curr Opin Microbiol. 2012;15(2):175–81. 10.1016/j.mib.2011.12.014 . 22265250

[11] Dorman CJ. H-NS, the genome sentinel. Nat Rev Microbiol. 2007;5(2):157–61. 10.1038/nrmicro1598 . 17191074

[12] Grainger DC. Structure and function of bacterial H-NS protein. Biochem Soc Trans. 2016;44(6):1561–9. 10.1042/BST20160190 . 27913665

[13] Singh SS, Singh N, Bonocora RP, Fitzgerald DM, Wade JT, Grainger DC. Widespread suppression of intragenic transcription initiation by H-NS. Genes Dev. 2014;28(3):214–9. 10.1101/gad.234336.113 . 24449106 PMC3923964

[14] Boudvillain M, Figueroa-Bossi N, Bossi L. Terminator still moving forward: expanding roles for Rho factor. Curr Opin Microbiol. 2013;16:118–24. 10.1016/j.mib.2012.12.003 23347833

[15] Ray-Soni A, Bellecourt MJ, Landick R. Mechanisms of Bacterial Transcription Termination: All Good Things Must End. Annu Rev Biochem. 2016;85:319–47. 10.1146/annurev-biochem-060815-014844 . 27023849

[16] Alifano P, Rivellini F, Limauro D, Bruni CB, Carlomagno MS. A consensus motif common to all Rho-dependent prokaryotic transcription terminators. Cell. 1991;64(3):553–63. 10.1016/0092-8674(91)90239-u . 1703923

[17] Di Salvo M, Puccio S, Peano C, Lacour S, Alifano P. RhoTermPredict: an algorithm for predicting Rho-dependent transcription terminators based on Escherichia coli, Bacillus subtilis and Salmonella enterica databases. BMC Bioinformatics. 2019;20(1):117 10.1186/s12859-019-2704-x . 30845912 PMC6407284

[18] Nadiras C, Eveno E, Schwartz A, Figueroa-Bossi N, Boudvillain M. A multivariate prediction model for Rho-dependent termination of transcription. Nucleic Acids Res. 2018;46(16):8245–60. 10.1093/nar/gky563 . 29931073 PMC6144790

[19] Bossi L, Schwartz A, Guillemardet B, Boudvillain M, Figueroa-Bossi N. A role for Rho-dependent polarity in gene regulation by a noncoding small RNA. Genes Dev. 2012;26(16):1864–73. 10.1101/gad.195412.112 . 22895254 PMC3426764

[20] Richardson JP, Grimley C, Lowery C. Transcription termination factor rho activity is altered in Escherichia coli with suA gene mutations. Proc Natl Acad Sci U S A. 1975;72(5):1725–8. 10.1073/pnas.72.5.1725 . 1098042 PMC432618

[21] Peters JM, Mooney RA, Kuan PF, Rowland JL, Keles S, Landick R. Rho directs widespread termination of intragenic and stable RNA transcription. Proc Natl Acad Sci U S A. 2009;106(36):15406–11. 10.1073/pnas.0903846106 . 19706412 PMC2741264

[22] Peters JM, Mooney RA, Grass JA, Jessen ED, Tran F, Landick R. Rho and NusG suppress pervasive antisense transcription in Escherichia coli. Genes Dev. 2012;26(23):2621–33. 10.1101/gad.196741.112 . 23207917 PMC3521622

[23] Saxena S, Gowrishankar J. Modulation of Rho-dependent transcription termination in Escherichia coli by the H-NS family of proteins. J Bacteriol. 2011;193(15):3832–41. 10.1128/JB.00220-11 . 21602341 PMC3147512

[24] Chandraprakash D, Seshasayee AS. Inhibition of factor-dependent transcription termination in Escherichia coli might relieve xenogene silencing by abrogating H-NS-DNA interactions in vivo. J Biosci. 2014;39(1):53–61. 10.1007/s12038-014-9413-4 . 24499790

[25] Boudreau BA, Hron DR, Qin L, van der Valk RA, Kotlajich MV, Dame RT, et al StpA and Hha stimulate pausing by RNA polymerase by promoting DNA-DNA bridging of H-NS filaments. Nucleic Acids Res. 2018;46(11):5525–46. 10.1093/nar/gky265 . 29718386 PMC6009659

[26] Kotlajich MV, Hron DR, Boudreau BA, Sun Z, Lyubchenko YL, Landick R. Bridged filaments of histone-like nucleoid structuring protein pause RNA polymerase and aid termination in bacteria. Elife. 2015;4 10.7554/eLife.04970 . 25594903 PMC4337669

[27] Tomar SK, Artsimovitch I. NusG-Spt5 proteins-Universal tools for transcription modification and communication. Chem Rev. 2013;113(11):8604–19. 10.1021/cr400064k . 23638618 PMC4259564

[28] Herbert KM, Zhou J, Mooney RA, Porta AL, Landick R, Block SM. E. coli NusG inhibits backtracking and accelerates pause-free transcription by promoting forward translocation of RNA polymerase. J Mol Biol. 2010;399(1):17–30. 10.1016/j.jmb.2010.03.051 . 20381500 PMC2875378

[29] Turtola M, Belogurov GA. NusG inhibits RNA polymerase backtracking by stabilizing the minimal transcription bubble. Elife. 2016;5 10.7554/eLife.18096 . 27697152 PMC5100998

[30] Burns CM, Nowatzke WL, Richardson JP. Activation of Rho-dependent transcription termination by NusG. Dependence on terminator location and acceleration of RNA release. J Biol Chem. 1999;274(8):5245–51. 10.1074/jbc.274.8.5245 . 9988775

[31] Li J, Mason SW, Greenblatt J. Elongation factor NusG interacts with termination factor rho to regulate termination and antitermination of transcription. Genes Dev. 1993;7(1):161–72. 10.1101/gad.7.1.161 . 8422985

[32] Sullivan SL, Gottesman ME. Requirement for E. coli NusG protein in factor-dependent transcription termination. Cell. 1992;68(5):989–94. 10.1016/0092-8674(92)90041-a . 1547498

[33] Burmann BM, Schweimer K, Luo X, Wahl MC, Stitt BL, Gottesman ME, et al A NusE:NusG complex links transcription and translation. Science. 2010;328(5977):501–4. 10.1126/science.1184953 . 20413501

[34] Lawson MR, Ma W, Bellecourt MJ, Artsimovitch I, Martin A, Landick R, et al Mechanism for the Regulated Control of Bacterial Transcription Termination by a Universal Adaptor Protein. Mol Cell. 2018;71(6):911–22. 10.1016/j.molcel.2018.07.014 . 30122535 PMC6151137

[35] Mooney RA, Schweimer K, Rosch P, Gottesman M, Landick R. Two structurally independent domains of E. coli NusG create regulatory plasticity via distinct interactions with RNA polymerase and regulators. J Mol Biol. 2009;391(2):341–58. 10.1016/j.jmb.2009.05.078 . 19500594 PMC2763281

[36] Saxena S, Myka KK, Washburn R, Costantino N, Court DL, Gottesman ME. Escherichia coli transcription factor NusG binds to 70S ribosomes. Mol Microbiol. 2018;108(5):495–504. 10.1111/mmi.13953 . 29575154 PMC5980749

[37] McGary K, Nudler E. RNA polymerase and the ribosome: the close relationship. Curr Opin Microbiol. 2013;16(2):112–7. 10.1016/j.mib.2013.01.010 23433801 PMC4066815

[38] Valabhoju V, Agrawal S, Sen R. Molecular Basis of NusG-mediated Regulation of Rho-dependent Transcription Termination in Bacteria. J Biol Chem. 2016;291(43):22386–403. 10.1074/jbc.M116.745364 . 27605667 PMC5077180

[39] Shashni R, Qayyum MZ, Vishalini V, Dey D, Sen R. Redundancy of primary RNA-binding functions of the bacterial transcription terminator Rho. Nucleic Acids Res. 2014;42(15):9677–90. 10.1093/nar/gku690 . 25081210 PMC4150792

[40] Bustamante VH, Martinez LC, Santana FJ, Knodler LA, Steele-Mortimer O, Puente JL. HilD-mediated transcriptional cross-talk between SPI-1 and SPI-2. Proc Natl Acad Sci U S A. 2008;105(38):14591–6. 10.1073/pnas.0801205105 . 18799744 PMC2567235

[41] Ellermeier CD, Ellermeier JR, Slauch JM. HilD, HilC and RtsA constitute a feed forward loop that controls expression of the SPI1 type three secretion system regulator hilA in Salmonella enterica serovar Typhimurium. Mol Microbiol. 2005;57(3):691–705. 10.1111/j.1365-2958.2005.04737.x . 16045614

[42] Golubeva YA, Sadik AY, Ellermeier JR, Slauch JM. Integrating global regulatory input into the Salmonella pathogenicity island 1 type III secretion system. Genetics. 2012;190(1):79–90. 10.1534/genetics.111.132779 . 22021388 PMC3249375

[43] Smith C, Stringer AM, Mao C, Palumbo MJ, Wade JT. Mapping the Regulatory Network for Salmonella enterica Serovar Typhimurium Invasion. MBio. 2016;7(5). 10.1128/mBio.01024-16 . 27601571 PMC5013294

[44] Chalissery J, Banerjee S, Bandey I, Sen R. Transcription termination defective mutants of Rho: role of different functions of Rho in releasing RNA from the elongation complex. J Mol Biol. 2007;371(4):855–72. 10.1016/j.jmb.2007.06.013 . 17599352 PMC1950744

[45] Figueroa-Bossi N, Schwartz A, Guillemardet B, D'Heygere F, Bossi L, Boudvillain M. RNA remodeling by bacterial global regulator CsrA promotes Rho-dependent transcription termination. Genes Dev. 2014;28(11):1239–51. 10.1101/gad.240192.114 . 24888591 PMC4052769

[46] Matsumoto Y, Shigesada K, Hirano M, Imai M. Autogenous regulation of the gene for transcription termination factor rho in Escherichia coli: localization and function of its attenuators. J Bacteriol. 1986;166(3):945–58. 10.1128/jb.166.3.945-958.1986 . 2423505 PMC215217

[47] Dillon SC, Espinosa E, Hokamp K, Ussery DW, Casadesús J, Dorman CJ. LeuO is a global regulator of gene expression in Salmonella enterica serovar Typhimurium. Mol Microbiol. 2012;85(6):1072–89. 10.1111/j.1365-2958.2012.08162.x . 22804842

[48] Espinosa E, Casadesús J. Regulation of Salmonella enterica pathogenicity island 1 (SPI-1) by the LysR-type regulator LeuO. Mol Microbiol. 2014;91(6):1057–69. 10.1111/mmi.12500 . 24354910

[49] Chen CC, Chou MY, Huang CH, Majumder A, Wu HY. A cis-spreading nucleoprotein filament is responsible for the gene silencing activity found in the promoter relay mechanism. J Biol Chem. 2005;280(6):5101–12. 10.1074/jbc.M411840200 . 15582999

[50] Fang M, Wu HY. A promoter relay mechanism for sequential gene activation. J Bacteriol. 1998;180(3):626–33. . 9457867 10.1128/jb.180.3.626-633.1998 PMC106931

[51] Nagarajavel V, Madhusudan S, Dole S, Rahmouni AR, Schnetz K. Repression by binding of H-NS within the transcription unit. J Biol Chem. 2007;282(32):23622–30. 10.1074/jbc.M702753200 . 17569663

[52] Rangarajan AA, Schnetz K. Interference of transcription across H-NS binding sites and repression by H-NS. Mol Microbiol. 2018;108(3):226–39. 10.1111/mmi.13926 29424946

[53] Buels R, Yao E, Diesh CM, Hayes RD, Munoz-Torres M, Helt G, et al JBrowse: a dynamic web platform for genome visualization and analysis. Genome Biol. 2016;17:66 10.1186/s13059-016-0924-1 . 27072794 PMC4830012

[54] Lithgow JK, Haider F, Roberts IS, Green J. Alternate SlyA and H-NS nucleoprotein complexes control hlyE expression in Escherichia coli K-12. Mol Microbiol. 2007;66(3):685–98. 10.1111/j.1365-2958.2007.05950.x . 17892462 PMC2156107

[55] Will WR, Bale DH, Reid PJ, Libby SJ, Fang FC. Evolutionary expansion of a regulatory network by counter-silencing. Nat Commun. 2014;5:5270 10.1038/ncomms6270 . 25348042 PMC4215172

[56] Bidnenko V, Nicolas P, Grylak-Mielnicka A, Delumeau O, Auger S, Aucouturier A, et al Termination factor Rho: From the control of pervasive transcription to cell fate determination in Bacillus subtilis. PLoS Genet. 2017;13(7):e1006909 10.1371/journal.pgen.1006909 . 28723971 PMC5540618

[57] Botella L, Vaubourgeix J, Livny J, Schnappinger D. Depleting Mycobacterium tuberculosis of the transcription termination factor Rho causes pervasive transcription and rapid death. Nat Commun. 2017;8:14731 10.1038/ncomms14731 . 28348398 PMC5379054

[58] Mäder U, Nicolas P, Depke M, Pane-Farre J, Debarbouille M, van der Kooi-Pol MM, et al Staphylococcus aureus Transcriptome Architecture: From Laboratory to Infection-Mimicking Conditions. PLoS Genet. 2016;12(4):e1005962 10.1371/journal.pgen.1005962 . 27035918 PMC4818034

[59] Pani B, Banerjee S, Chalissery J, Muralimohan A, Loganathan RM, Suganthan RB, et al Mechanism of inhibition of Rho-dependent transcription termination by bacteriophage P4 protein Psu. J Biol Chem. 2006;281(36):26491–500. 10.1074/jbc.M603982200 . 16829521 PMC1950596

[60] Mayer A, Landry HM, Churchman LS. Pause & go: from the discovery of RNA polymerase pausing to its functional implications. Curr Opin Cell Biol. 2017;46:72–80. 10.1016/j.ceb.2017.03.002 . 28363125 PMC5505790

[61] Jain S, Gupta R, Sen R. Rho-dependent transcription termination in bacteria recycles RNA polymerases stalled at DNA lesions. Nat Commun. 2019;10(1):1207 10.1038/s41467-019-09146-5 . 30872584 PMC6418286

[62] Kang JY, Mooney RA, Nedialkov Y, Saba J, Mishanina TV, Artsimovitch I, et al Structural Basis for Transcript Elongation Control by NusG Family Universal Regulators. Cell. 2018;173(7):1650–62. 10.1016/j.cell.2018.05.017 . 29887376 PMC6003885

[63] Lilleengen K. Typing of *Salmonella typhimurium* by means of bacteriophage. Acta Pathol Microbiol Scand. 1948;77(Suppl):2–125. 10.1111/j.1699-0463.1952.tb00174.x 14933053

[64] Figueroa-Bossi N, Coissac E, Netter P, Bossi L. Unsuspected prophage-like elements in Salmonella typhimurium. Mol Microbiol. 1997;25(1):161–73. 10.1046/j.1365-2958.1997.4451807.x . 11902718

[65] Bertani G. Lysogeny at mid-twentieth century: P1, P2, and other experimental systems. J Bacteriol. 2004;186(3):595–600. 10.1128/JB.186.3.595-600.2004 . 14729683 PMC321500

[66] Maloy SR, Roth JR. Regulation of proline utilization in Salmonella typhimurium: characterization of put::Mu d(Ap, lac) operon fusions. J Bacteriol. 1983;154(2):561–8. . 6302076 10.1128/jb.154.2.561-568.1983 PMC217501

[67] Schmieger H. Phage P22-mutants with increased or decreased transduction abilities. Mol Gen Genet. 1972;119(1):75–88. 10.1007/bf00270447 . 4564719

[68] Datsenko KA, Wanner BL. One-step inactivation of chromosomal genes in Escherichia coli K-12 using PCR products. Proc Natl Acad Sci U S A. 2000;97(12):6640–5. 10.1073/pnas.120163297 . 10829079 PMC18686

[69] Uzzau S, Figueroa-Bossi N, Rubino S, Bossi L. Epitope tagging of chromosomal genes in Salmonella. Proc Natl Acad Sci U S A. 2001;98(26):15264–9. 10.1073/pnas.261348198 . 11742086 PMC65018

[70] Reznikoff WS. Transposon Tn5. Annu Rev Genet. 2008;42:269–86. 10.1146/annurev.genet.42.110807.091656 . 18680433

[71] Macconkey A. Lactose-Fermenting Bacteria in Faeces. J Hyg (Lond). 1905;5(3):333–79. 10.1017/s002217240000259x . 20474229 PMC2236133

[72] Duncan MC, Forbes JC, Nguyen Y, Shull LM, Gillette RK, Lazinski DW, et al Vibrio cholerae motility exerts drag force to impede attack by the bacterial predator Bdellovibrio bacteriovorus. Nat Commun. 2018;9(1):4757 10.1038/s41467-018-07245-3 . 30420597 PMC6232129

[73] Figueroa-Bossi N, Lemire S, Maloriol D, Balbontín R, Casadesús J, Bossi L. Loss of Hfq activates the σ^E^-dependent envelope stress response in Salmonella enterica. Mol Microbiol. 2006;62(3):838–52. 10.1111/j.1365-2958.2006.05413.x . 16999834

[74] Miller JH. A Short Course in Bacterial Genetics. A Laboratory Manual and Handbook for Escherichia coli and Related Bacteria Cold Spring Harbor, New York: Cold Spring Harbor Laboratory Press; 1992.

[75] Thomsen ND, Berger JM. Running in reverse: the structural basis for translocation polarity in hexameric helicases. Cell. 2009;139(3):523–34. 10.1016/j.cell.2009.08.043 . 19879839 PMC2772833

[76] Skordalakes E, Berger JM. Structure of the Rho transcription terminator: mechanism of mRNA recognition and helicase loading. Cell. 2003;114(1):135–46. 10.1016/s0092-8674(03)00512-9 . 12859904

[77] T Robinson JT, Thorvaldsdottir H, Winckler W, Guttman M, Lander ES, Getz G, et al Integrative genomics viewer. Nat Biotechnol. 2011;29(1):24–6. 10.1038/nbt.1754 . 21221095 PMC3346182

