# Supplementary figures and images for: NusG prevents transcriptional invasion of H-NS-silenced genes
Source: PLoS Genet. 2019 Oct 7;15(10):e1008425. doi: 10.1371/journal.pgen.1008425 (PMC6797219; doi:10.1371/journal.pgen.1008425)

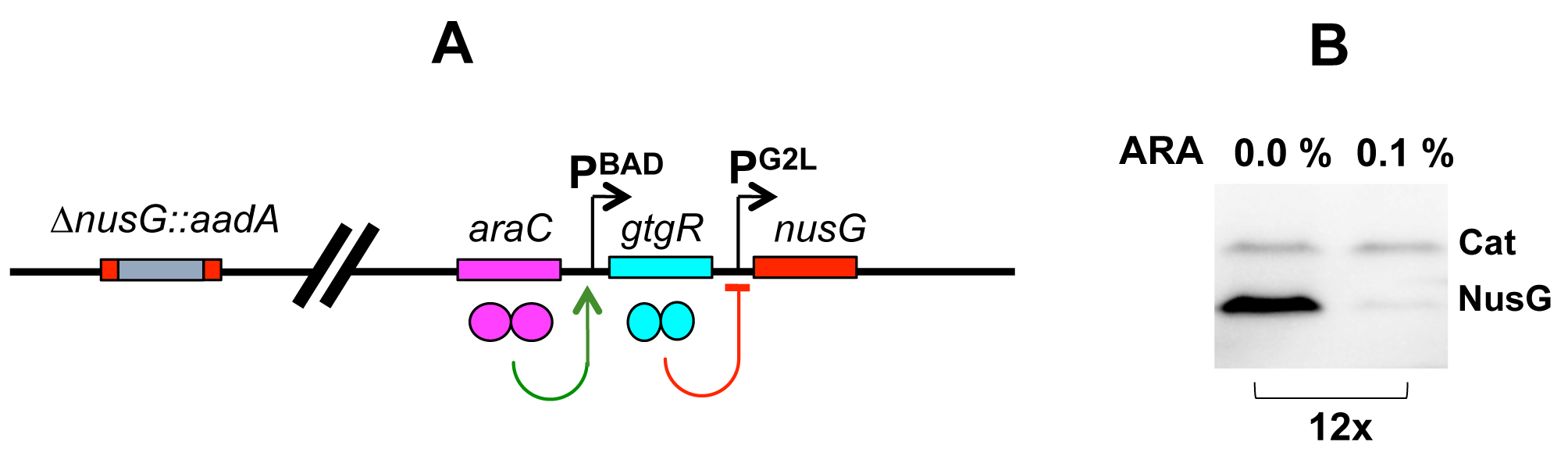

Supplement: S1 Fig — (A) Diagram showing the main features of the NusG depletable strain. This strain (MA12996) carries the Gifsy-2 prophage repressor gene (gtgR) under the control of the arabinose operon promoter (PBAD). Gifsy-2 left operon promoter (PG2L, lying on the 3’ side of gtgR) is fused to the coding sequence of nusG. The native copy of the nusG gene as well as the entirety of Gifsy-1 and Gifsy-2 prophages are deleted. (B) Western blot analysis of a strain (MA13953) carrying 3xFLAG epitope fusions to the 3’ end of the GtgR-repressible nusG and to the cat gene (as an internal standard) grown in the absence or in the presence of 0.1% arabinose. Values below the blot represent the fold change in the intensity of the NusG band before and after the ARA treatment, normalized to the intensity of the cat signal in the same lane. (TIF) [file pgen.1008425.s001.tif]

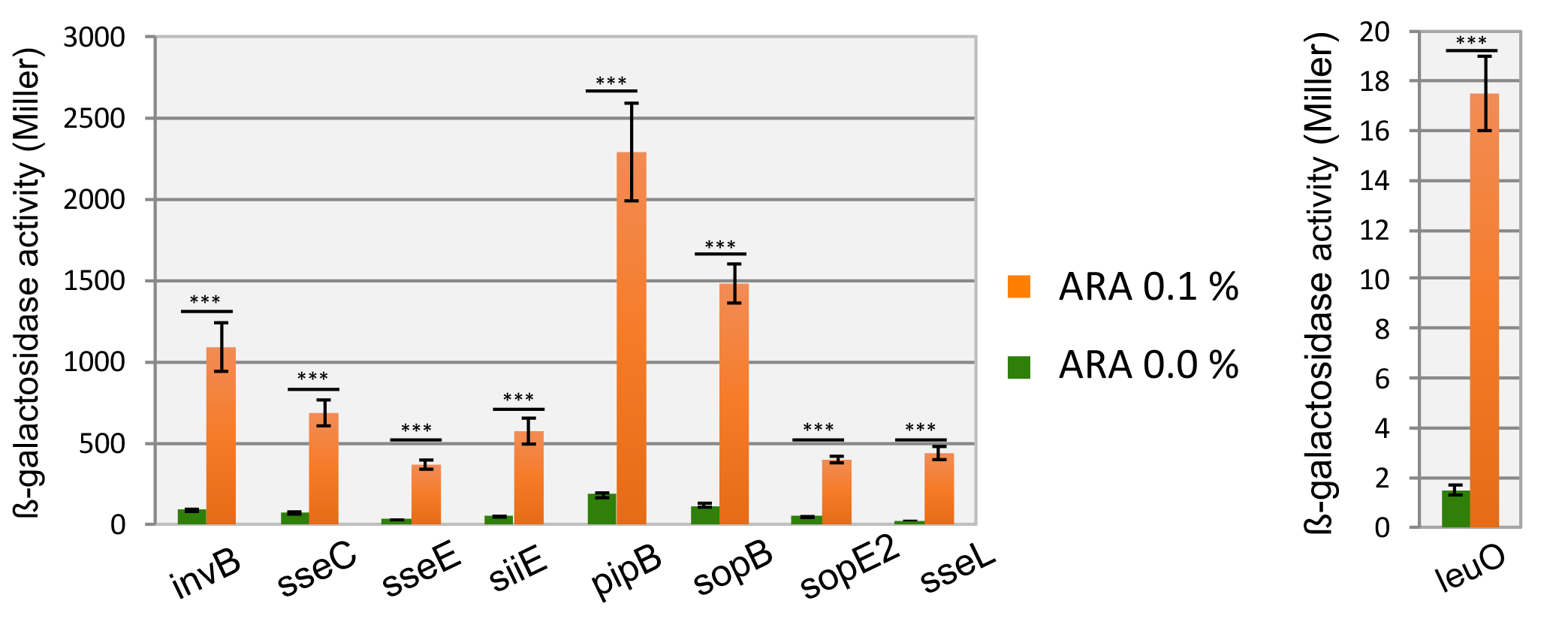

Supplement: S2 Fig — lacZ gene fusions to genes from pathogenicity islands or related loci were obtained following random lacZ transposition in a strain carrying the nusG gene under the control of an arabinose-inducible repressor. Strains carrying the various fusions were grown in the presence or absence of arabinose to early stationary phase (OD600 = 2–3.5) and assayed for ß-galactosidase activity (two separate assays, each performed on two independent cultures). Statistical significance was calculated using unpaired two-tailed Student’s T (***, P < 0.001). (TIF) [file pgen.1008425.s002.tif]

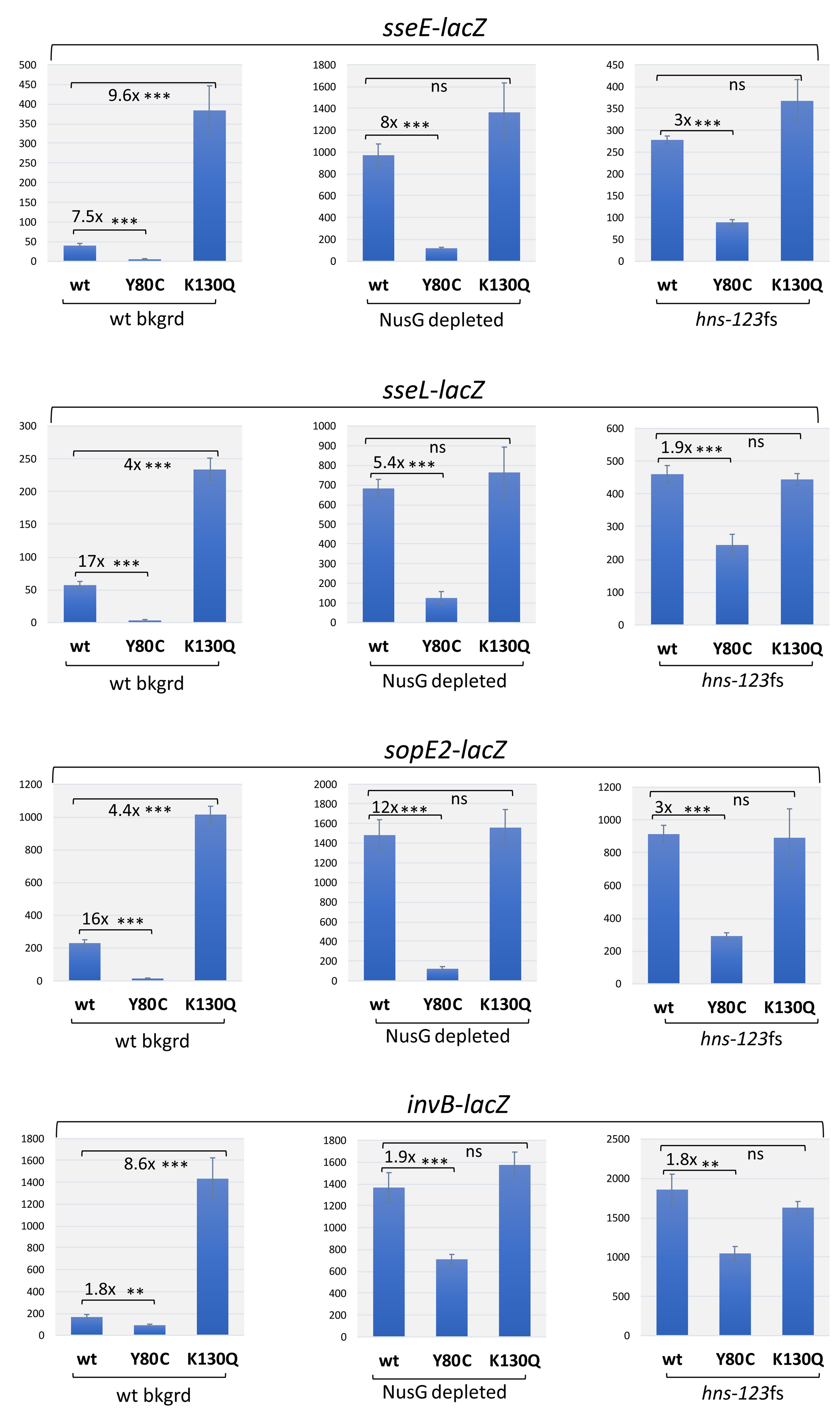

Supplement: S3 Fig — Strains carrying wild-type or mutant alleles of rho (rho Y80C or K130Q) in combination with either a wild-type or an ARA-repressible version of the nusG gene, or with either wild-type or mutant hns (hns-123fs), and one of four different lacZ fusions identified in this study, were grown to early stationary phase and assayed for ß-galactosidase activity as described in Materials and methods. Assays were performed at least twice, each time with two biological replicates. Statistical significance was calculated by the Student’s T test (unpaired two-tailed; ***, P < 0.001; **, P < 0.01; *, P < 0.05; ns, P > 0.05). Values in vertical axes represent Miller units of ß-galactosidase activity [74]. (TIF) [file pgen.1008425.s003.tif]

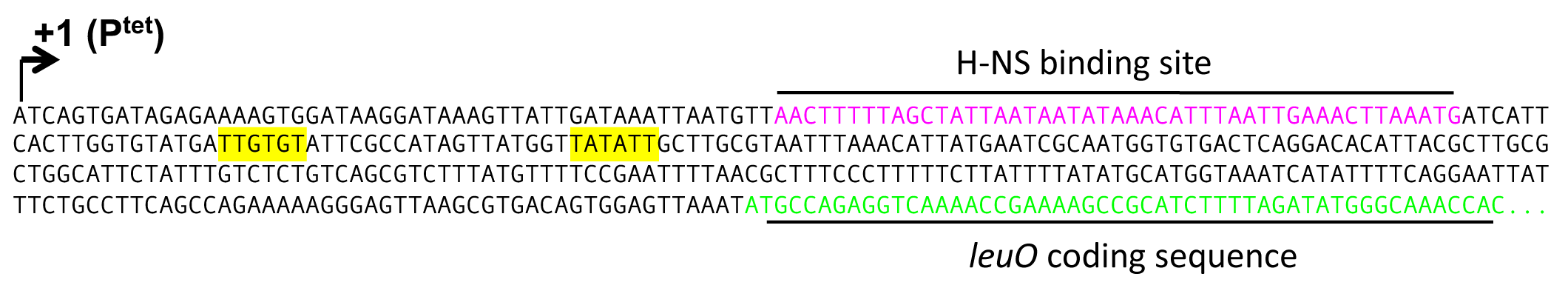

Supplement: S4 Fig — The leuO promoter sequence (yellow boxes) and the H-NS binding site (purple lettering) are from refs [49] and [50], respectively. (TIF) [file pgen.1008425.s004.tif]

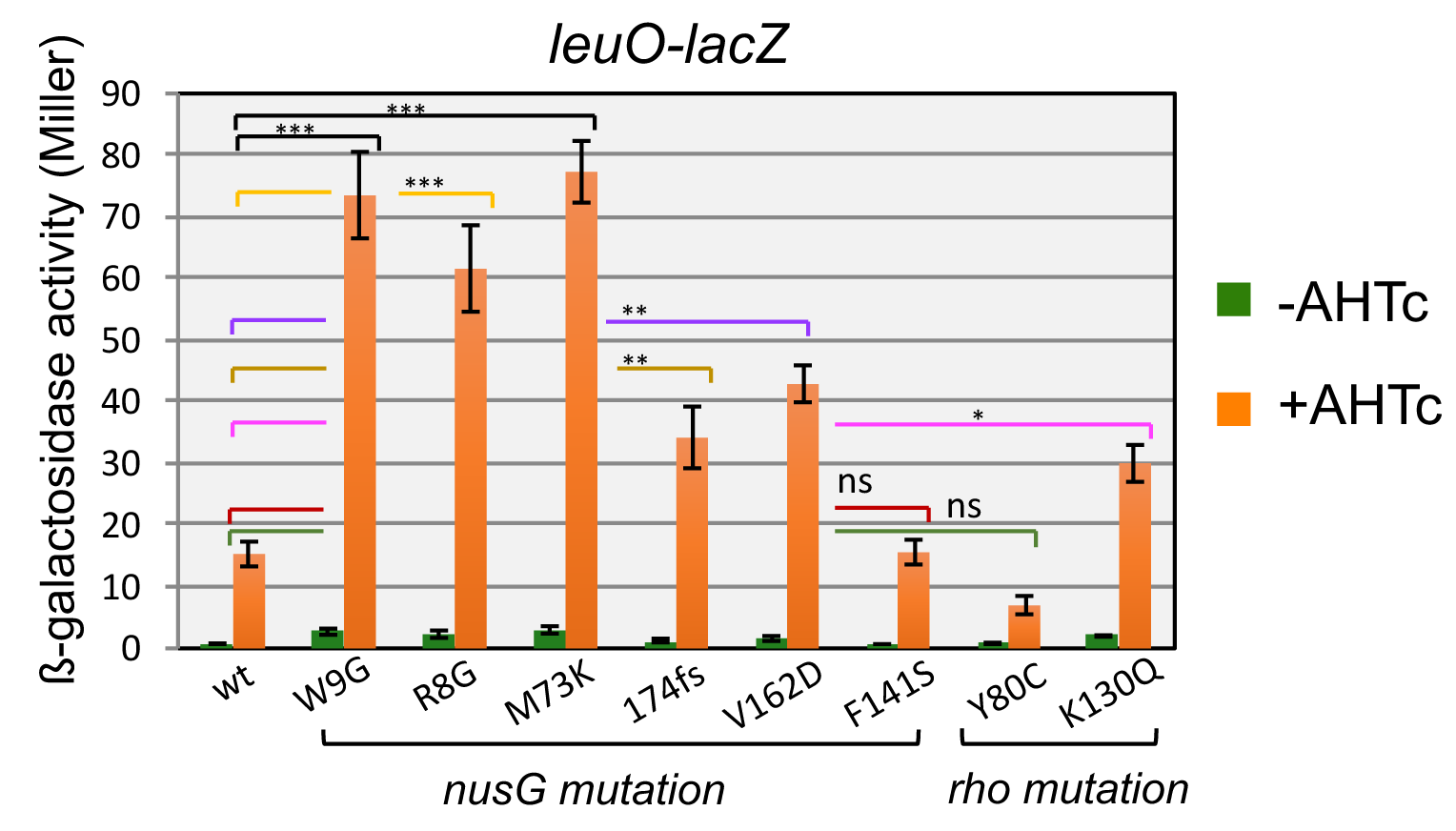

Supplement: S5 Fig — A strain carrying Ptet-leuO-lacZ is phenotypically Lac- in the presence of the Ptet inducer (AHTc), because H-NS prevents transcription from reaching the leuO coding sequence. NusG NTD mutations W9G, R8G and M73K were isolated selecting Lac+ derivatives. NusG CTD mutations 174fs, V162D and F141S and Rho mutations Y80C and K130K were isolated previously (see main text). Strains carrying these different alleles were grown in the presence or absence of AHTc (0.4 μg/ml) to early stationary phase and assayed for ß-galactosidase activity (two independent assays, with two biological replicas each). Statistical significance of each mutant versus wild-type difference in AHTc-supplemented cultures was calculated by the Student’s T test (***, P < 0.001; **, P < 0.01; ns, P > 0.05). Results show that NusG NTD mutations are significantly more effective than NusG NTD and Rho mutations at relieving the H-NS block. Although some of the latter do cause some increase in lac expression, the increase is not sufficient to render the strain Lac+. (TIF) [file pgen.1008425.s005.tif]

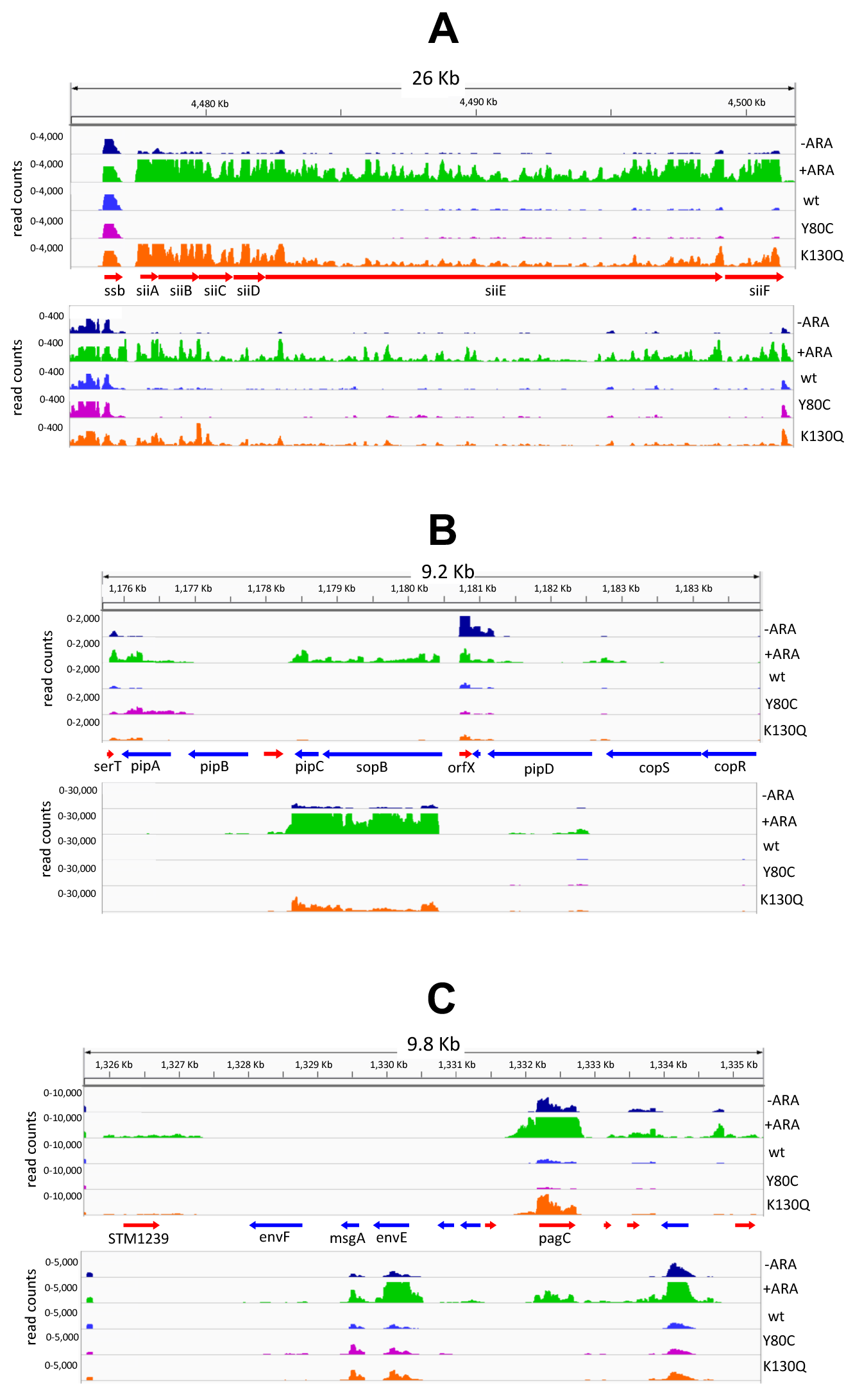

Supplement: S6 Fig — Profiles above and below arrows correspond to sense transcription of right-oriented genes (red arrows) and left-oriented genes (blue arrows), respectively. (A) SPI-4; (B) SPI-5; (C) SPI-11. Note the increase of anti-sense transcription throughout SPI-4 (A), in the pipC-sopB region of SPI-5 (B) and in the pagC locus (C) during NusG depletion. (TIF) [file pgen.1008425.s006.tif]

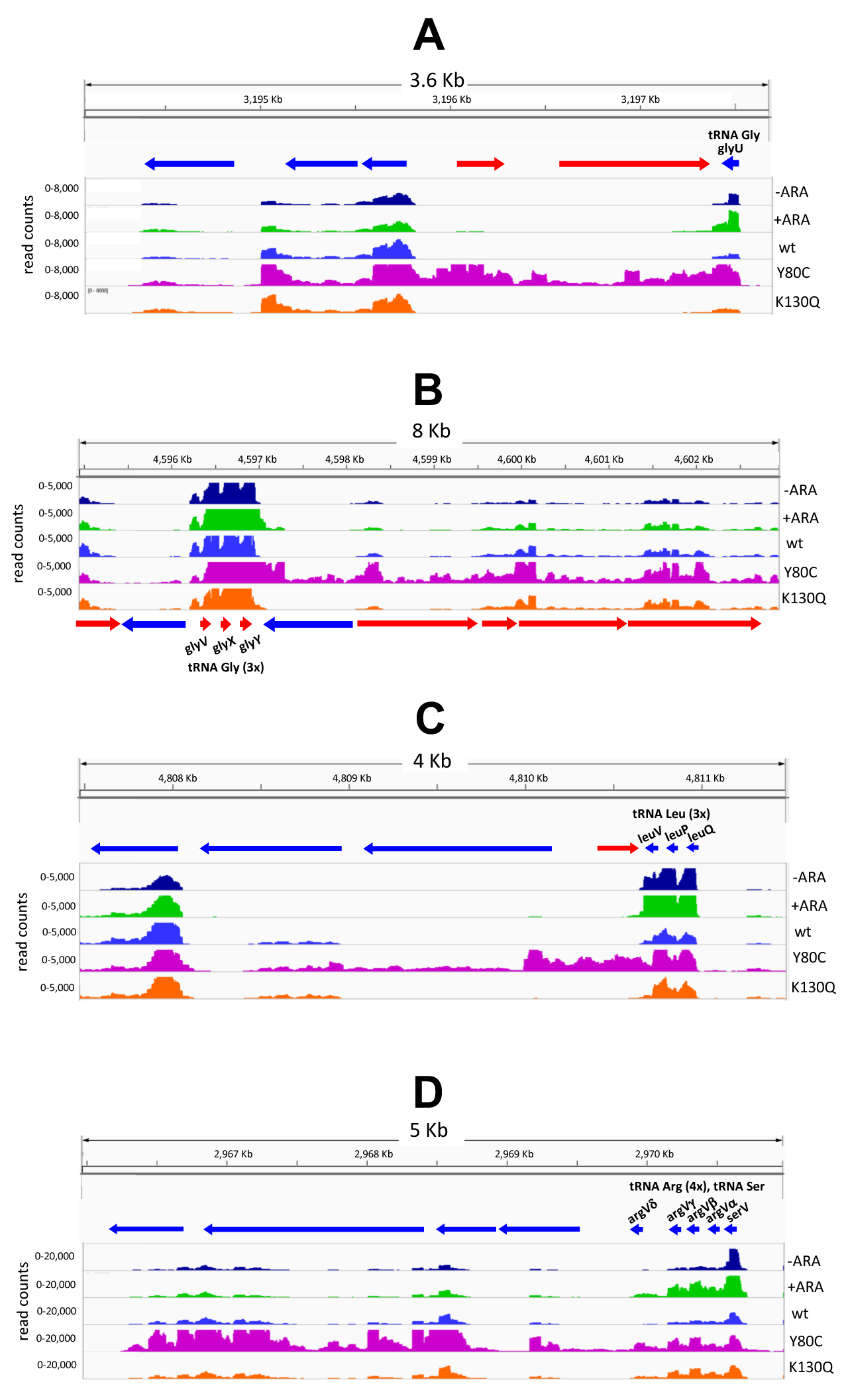

Supplement: S7 Fig — Profiles above arrows correspond to sense transcription of right-oriented genes (red arrows); profiles below arrows correspond to sense transcription of left-oriented genes (blue arrows). (A) glyU; (B) glyV glyX glyY; (C) leuV, leuP, leuQ; (D) serV argVα argVβ argVγ argVδ. (TIF) [file pgen.1008425.s007.tif]

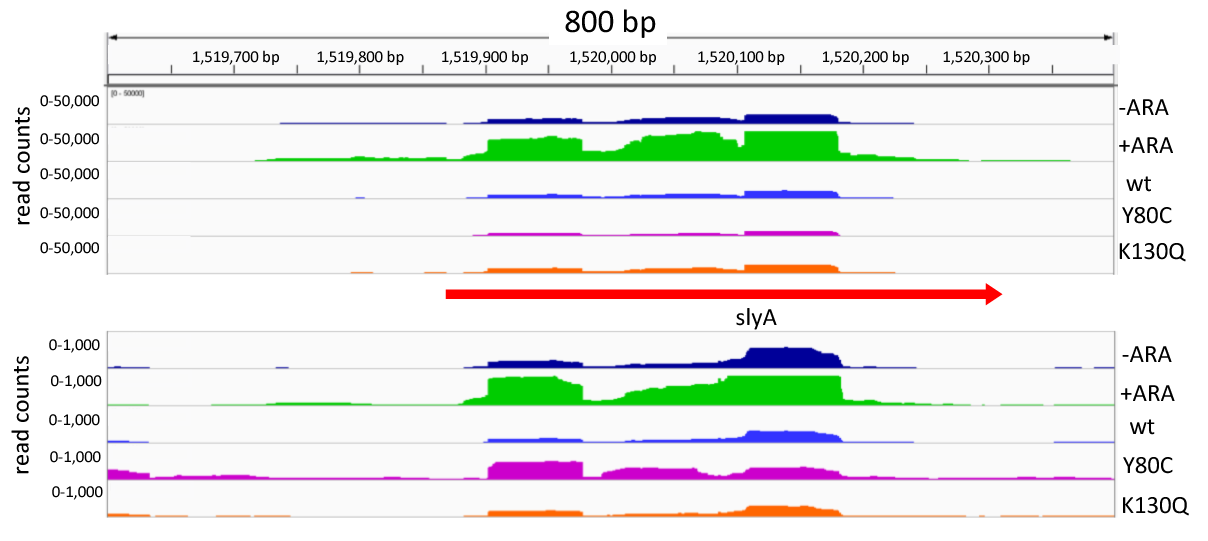

Supplement: S8 Fig — Profiles above the red arrow correspond to slyA sense transcription; profiles below the arrow correspond to slyA anti-sense transcription. (TIF) [file pgen.1008425.s008.tif]
