# Supplementary material for: NusG prevents transcriptional invasion of H-NS-silenced genes
Source: PLoS Genet. 2019 Oct 7;15(10):e1008425. doi: 10.1371/journal.pgen.1008425 (PMC6797219; doi:10.1371/journal.pgen.1008425)
Supplement: S1 Methods — (DOCX) [file pgen.1008425.s017.docx]

**Supplementary Methods**

**Construction of a NusG-depletable strain**

The making of this strain (MA12996) involved assembling modules constructed separately by λ-*red* recombineering and phage P22 transduction.

**1. *araC*-P^BAD^*gtgR*-P^G2L^ (strain MA11589).** The first step was to construct strain MA11330. In this strain, a *tet* cassette (*tetR*-*tetA* genes, amplified with oligonucleotides ppK65 and ppK66 as primers) replaces the entirety of the *Salmonella araBAD* operon (starting from *araB* initiation codon to a position in the middle of *araD*). Using *tet*-associated fusaric acid-sensitivity as counter-selection [1], the *tet* cassette was exchanged with a PCR-amplified fragment encompassing phage Gifsy-2 repressor gene (*gtgR*) and the adjacent P^G2L^ promoter [2]. The primers used for *gtgR*-P^G2L^ amplification (ppM27-ppM28) were designed to place the *gtgR* initiation codon at the exact position normally occupied by *araB* AUG (11558). The resulting construct was moved to a strain cured for both Gifsy-1 and Gifsy-2 prophages by P22 transduction.

**2. *nusG cat* (strain MA11111).** This strain was constructed previously by placing the *cat* gene (amplified with primers ppJ92 and ppJ93) on the 3’ side of the *nusG* gene [3]. In the final construct, the *cat* open reading frame (including the Shine-Dalgarno motif) is positioned 20 bp downstream from the end of the *nusG* coding sequence and is expressed from the *secE-nusG* promoter.

**3. *araC*-P^BAD^*gtgR*-P^G2L^*nusG* *cat* (strain MA12946).** The *nusG-cat* segment was amplified from the chromosome of strain MA11111 (with primers ppX62 and ppL15) and placed next to the P^G2L^ promoter in strain MA11589 (above). The resulting strain, MA12946, has two functional copies of the *nusG* gene, one at its natural location, the other at the *ara* locus under the control of the arabinose-inducible GtgR repressor.

**4. *araC*-P^BAD^*gtgR*-P^G2L^*nusG* *cat* ∆*nusG*::*aadA* (strain 12996).** The *aadA* gene for spectinomycin-resistance was PCR-amplified from plasmid pSEB5 [4] with oligonucleotides ppI98 and ppI99 and used to delete the native copy of *nusG* in strain 12996. The resulting strain, MA12996, undergoes NusG depletion when grown in the presence of arabinose.

**Placing a *tetR*-P^tet^ cassette upstream of the *leuO* gene**

A tripartite module comprising a *tetR*-P^tet^ cassette, a *ccdB* toxin gene fused to P^tet^, and a *cat* gene (*tetR*-P^tet^*ccdB cat*) was amplified from plasmid pDLM3 with oligonucleotides ppAC19 and ppAC20 and inserted 651 bp upstream of the initiation codon of *leuO* selecting for chloramphenicol-resistance. This step was carried out independently with strains MA3409 (*leuO*^wt^) and MA13672 (*leuO*-*lacZY*). The resulting strains (MA13705 and MA13682, respectively) are sensitive to anhydrotetracycline (AHTc), which activates P^tet^, leading to *ccdB* expression. Using AHTc-sensitivity as counter selection, the *ccdB cat* portion of the construct was replaced by a 91 bp DNA fragment (produced by “fill-in” PCR with oligonucleotides ppAC75 and ppAC76) designed to place the + 1 position of P^tet^-directed transcription 345 bp upstream from the beginning of the *leuO* orf. The resulting strains, with alleles P^tet^*345*-*leuO* and P^tet^345-*leuO*-*lacZY* are named MA13710 and MA13700, respectively.

**Placing the *tetR*-P^tet^ cassette upstream of the *proU* operon**

The procedure was similar to that followed for *leuO*, expect that the tripartite module with the counter-selectable marker (*ccdB*) carried a *kan* gene in the place of *cat*. The module was amplified from plasmid pDLM9 with oligonucleotides ppAG56 and ppAG57 and inserted at a position 352 bp upstream of the beginning of the *proV* orf in a wild-type strain (MA3409). The *ccdB-kan* portion of the module was subsequently replaced by a 113 bp DNA fragment (produced by “fill-in” PCR with oligonucleotides ppAG58 and ppAG59) selecting AHTc-resistance. This step placed the + 1 position of P^tet^-directed transcription 378 bp upstream from the beginning of the *proV* orf. The resulting strain, MA14216 (relevant allele: P^tet^378-*proV*), was used for the construction of 3xFLAG-tagged and *lacZ* fusion derivatives of *proV* (see below).

**Construction of 3xFLAG-tagged and *lacZ*-fusion derivatives of P^tet^*345*-*leuO***

The sequence encoding the 3xFLAG epitope was fused in frame to the last codon of *leuO* in the P^tet^*345*-*leuO* background using a fragment amplified from plasmid pSUB11 with primers ppAC81-ppAC82 as previously described [5]. The strain obtained, MA13715, was subsequently modified by the addition of *cat*-1xFLAG, yielding strain MA13834. A new *lacZ* fusion to P^tet^*345*-*leuO* was constructed by the method of Ellermeier and coworkers [6]. Briefly, an FRT-*kan*-FRT cassette amplified from plasmid pKD13 [7] with primers ppAD48 and ppAD15 was placed near the 5’ end of *leuO*. Transformation of the resulting strain with Flp recombinase plasmid pCP20 [8] allowed the excision of *kan* cassette and subsequent Flp-mediated integration of plasmid pCE40 [6], generating fusion P^tet^*345*-*leuO-*FRT-*lacZY* (strain 13772). Unlike the P^tet^*345*-*leuO*-*lacZ* fusion described above (obtained by *lacZY* transposition), which carries the *lacZ* gene at position 266 of the 945 bp *leuO* gene, P^tet^ *345*-*leuO-*FRT-*lacZY* has the fusion junction closer to the beginning of *leuO* (position 11).

**Construction of 3xFLAG-tagged and *lacZ*-fusion derivatives of P^tet^378-*proV***

The 3xFLAG-encoding sequence was fused to the last codon of *proV* (in the strain MA14216) using a fragment amplified from plasmid pSUB11 [5] with primers ppAG63-ppAG64.

For the construction of a *proV-lacZ* fusion, the FRT-*kan*-FRT cassette of plasmid pKD13 [7], amplified with primers ppAG74 and ppAG62, was used to replace the segment of the *proV* orf from position +289 to a site immediately downstream from the end of the gene. Subsequent transformation with plasmid pCP20 allowed the sequential excision of the *kan* gene and the Flp-mediated integration of *pir*-dependent plasmid pPS2, a pCE40 derivative designed to generate transcriptional fusions. The resulting strain, MA14237 (P^tet^378-*proV289-lacZY*), was used as starting material for the experiments described in the text.

**Western blot analysis**

Whole cell bacterial lysates, prepared as described in the main text (see Materials and Methods) were separated by SDS-polyacrylamide gel electrophoresis (Acrylamide-Bisacrylamide 29:1; between 10% and 15% depending on the molecular weight of the proteins being analyzed). BioRad's Precision Plus Kaleidoscope molecular weight standards were included as migration markers. After the gel was run, proteins were electro-transferred to a PVDF membrane, which was blocked with PBS containing 5% powdered low-fat milk. For the detection of 3xFLAG tagged proteins, the blocking buffer was removed by washing with PBS. Membrane was exposed to primary anti-FLAG antibody (anti-FLAG M2 from Sigma-Aldrich) in a PBS solution for 2 hr and rinsed thoroughly in PBS 0.05% Tween 20 before the secondary antibody (Goat anti-mouse peroxidase-conjugated secondary antibodies; from Sigma-Aldrich). For Rho protein detection, polyclonal antibodies raised against Rho protein purified from *E. coli* were used. Secondary antibodies were goat anti-rabbit HRP conjugated antibodies (Invitrogen). Membranes were exposed to secondary antibodies in PBS solution for 1hr followed by thorough washing with PBS 0.05% Tween 20. Results were revealed with the Amersham ECL Select Western Blotting Detection Reagent (GE Healthcare) and imaged on the ChemiDoc Touch imaging system (BioRad). Bands were quantified with the ImageJ program.

**RNA-Seq**

RNA extracted as described in the main text (triplicate samples) was quality-controlled on an Agilent Bioanalyzer 2100, using RNA 6000 pico kit (Agilent Technologies). 2 µg of total RNA were treated with DNAse (Baseline Zero DNAse, Epicentre) prior to ribosomal RNA depletion using the RiboZero Bacteria magnetic Kit from Illumina, according to the manufacturer recommendations. Samples were then checked on the Agilent Bioanalyzer for proper rRNA depletion. Directional RNA-seq libraries were constructed using ScriptSeq V2 RNA-seq library preparation kit (Illumina), according to the manufacturer recommendations. Libraries were pooled in equimolar amounts and sequenced (Paired-end 2x75pb) on an Illumina NextSeq500 instrument, using a NextSeq 500 Mid Output 150 cycles kit. Demultiplexing was performed with the bcl2fastq2 V2.2.18.12 program and adapters were trimmed with Cutadapt1.15. Only reads longer than 10pb were kept. Reads were mapped on the *Salmonella enterica* serovar Typhimurium strain LT2 genome (GCA_000006945.2_ASM694v2_genomic.fna) with BWA 0.6.2-r126. BigWig files were generated with the bamCoverage 3.1.0 tool, from the deepTools python suite, to obtain a coverage track (number of reads per base). Mapped reads were assigned to genes with the featureCounts program. Differential expression analysis was performed with the DESeq2 1.18.1 software package.

Experimental designs for the study of the effects of NusG depletion and Rho mutations can be found at <https://www.ebi.ac.uk/arrayexpress/experiments/E-MTAB-8159> and <https://www.ebi.ac.uk/arrayexpress/experiments/E-MTAB-8206> , respectively.

**References for Supplementary Methods**

1. Figueroa-Bossi N, Bossi L. Recombineering applications for the mutational analysis of bacterial RNA-binding proteins and their sites of action. Methods Mol Biol. 2015;1259:103-16. PMID: 25579582.

2. Lemire S, Figueroa-Bossi N, Bossi L. A singular case of prophage complementation in mutational activation of recET orthologs in Salmonella enterica serovar Typhimurium. J Bacteriol. 2008;190(20):6857-66. PMID: 18689471.

3. Bossi L, Schwartz A, Guillemardet B, Boudvillain M, Figueroa-Bossi N. A role for Rho-dependent polarity in gene regulation by a noncoding small RNA. Genes Dev. 2012;26(16):1864-73. PMID: 22895254.

4. Figueroa-Bossi N, Lemire S, Maloriol D, Balbontín R, Casadesús J, Bossi L. Loss of Hfq activates the σ^E^-dependent envelope stress response in Salmonella enterica. Mol Microbiol. 2006;62(3):838-52. PubMed PMID: 16999834.

5. Uzzau S, Figueroa-Bossi N, Rubino S, Bossi L. Epitope tagging of chromosomal genes in Salmonella. Proc Natl Acad Sci U S A. 2001;98(26):15264-9. PMID: 11742086.

6. Ellermeier CD, Janakiraman A, Slauch JM. Construction of targeted single copy lac fusions using lambda Red and FLP-mediated site-specific recombination in bacteria. Gene. 2002;290(1-2):153-61. PMID: 12062810.

7. Datsenko KA, Wanner BL. One-step inactivation of chromosomal genes in Escherichia coli K-12 using PCR products. Proc Natl Acad Sci U S A. 2000;97(12):6640-5. PMID: 10829079.

8. Cherepanov PP, Wackernagel W. Gene disruption in Escherichia coli: TcR and KmR cassettes with the option of Flp-catalyzed excision of the antibiotic-resistance determinant. Gene. 1995;158(1):9-14. PMID: 7789817.
